# Propofol Modulates Fear Memory Reconsolidation by Inhibiting D2 Receptor‐Expressing Neurons in the Medial Prefrontal Cortex

**DOI:** 10.1002/advs.78527

**Published:** 2026-10-28

**Authors:** Jie Li, Yi‐Ge Guo, Xing Xu, Gui‐Lin Meng, Han Li, Liu‐Wei Chu, Xin‐Yuan Chen, Xiao‐Yun Guo, Tao Xu, Ti‐Fei Yuan, Ji Hu, Xiao‐Na Zhu

**Affiliations:** ^1^ Shanghai Key Laboratory of Mental Disorders Translational Research Brain Health Institute National Center for Mental Disorders Shanghai Mental Health Center Shanghai Jiao Tong University School of Medicine and School of Psychology Shanghai China; ^2^ School of Life Science and Technology ShanghaiTech University Shanghai China; ^3^ Department of Psychiatry The Affiliated Hospital of Guizhou Medical University Guiyang Guizhou China; ^4^ Shanghai Sixth People's Hospital Shanghai Jiao Tong University School of Medicine Shanghai China; ^5^ Department of Neurology Tongren Hospital Shanghai Jiao Tong University School of Medicine Shanghai China

**Keywords:** D2 receptor‐expressing neurons, D2 receptors, fear memory reconsolidation, medial prefrontal cortex (mPFC), mPFC^D2^‐BLA pathway, propofol

## Abstract

Fear memory reconsolidation provides a temporal window for modifying established fear memories, yet the underlying cellular and neural circuit mechanisms remain incompletely understood. Using in vivo fiber photometry and chemogenetic approaches in freely moving mice, we show that propofol modulates fear memory reconsolidation by inhibiting D2 receptor‐expressing neurons in the medial prefrontal cortex (mPFC). These neurons exhibit heightened activity during fear memory reconsolidation and project prominently to the basolateral amygdala (BLA). Notably, local pharmacological activation of D2 receptors in the mPFC recapitulates the fear‐attenuating effect of propofol when administered during the reconsolidation window. Circuit‐level investigations further show that propofol suppresses the reconsolidation‐associated enhancement of the mPFC^D2^‐BLA synaptic transmission, and chemogenetic activation of this projection counteracts the effect of propofol on fear memory reconsolidation. Moreover, selective knockdown of D2 receptors in BLA‐projecting mPFC neurons diminishes the effect of propofol, supporting a critical role for D2 receptor signaling within this projection‐defined circuit. Together, these findings identify the mPFC^D2^‐BLA circuit as a key neural substrate through which propofol modulates fear memory reconsolidation and highlight prefrontal D2 receptor signaling as a potential target for reconsolidation‐based interventions aimed at maladaptive fear memories.

## Graphical abstract

This study shows that propofol modulates fear‐memory reconsolidation in mice. It works by suppressing D2 receptor‐expressing neurons within the medial prefrontal cortex and their connections to the basolateral amygdala. D2‐receptor agonists reproduce this fear‐reducing effect, offering potential non‐anesthetic therapeutic strategies for trauma‐related fear disorders.

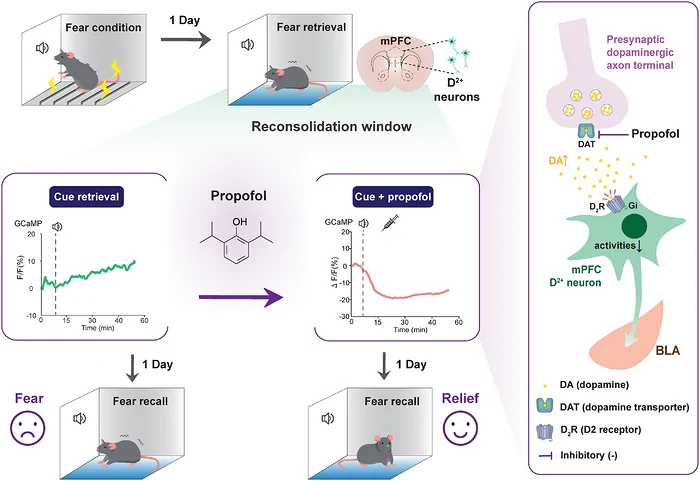

## 1 Introduction

Fear memory is a form of memory associated with traumatic events that can elicit fear responses and contribute to several intractable psychiatric disorders, including post‐traumatic stress disorder (PTSD), anxiety disorders, and specific phobias [1]. Previous studies have indicated that modifying maladaptive fear memories holds therapeutic potential for these disorders [2, 3]. Fear memory attenuation can be targeted during two distinct phases: memory reconsolidation and extinction. However, the clinical efficacy of extinction therapy, a widely used strategy for reducing pathological fear responses, is substantially constrained by the inherent fragility of extinction memories. These memories are susceptible to well‐established phenomena such as reinstatement, renewal, and spontaneous recovery [4, 5]. In contrast, recent evidence suggests that targeting fear memory reconsolidation may provide an alternative therapeutic opportunity to modify established maladaptive fear memories in a more persistent manner [3].

Fear memory reconsolidation refers to the process by which retrieved fear memories transiently return to a labile state and require new protein synthesis for their persistence [6, 7, 8, 9]. During this labile state, the memory is amenable to modification, including weakening or strengthening. Studies have shown that disrupting the reconsolidation process using protein synthesis inhibitors can prevent memory relapse [6]. However, the clinical translation of pharmacological approaches targeting fear memory reconsolidation is limited by the toxicity of commonly used agents, such as the protein synthesis inhibitor anisomycin, which precludes their safe use in humans [6, 10]. This limitation underscores the need to identify clinically applicable agents that can safely modulate reconsolidation and produce sustained attenuation of maladaptive fear memories.

Anesthetic agents are known to modulate memory formation and retrieval [11, 12]. Beyond these effects, growing evidence suggests that certain anesthetics, including propofol, can attenuate fear memories, raising the possibility that anesthetic‐sensitive mechanisms may be exploited to modify maladaptive fear memories [13, 14]. This possibility is further supported by reports demonstrating the neuropsychiatric effects of propofol in conditions such as depression and anxiety [15, 16]. Notably, recent clinical studies have shown that anesthetic agents can interfere with memory consolidation and reconsolidation, suggesting their potential utility for modulating fear memory reconsolidation [17]. Among these agents, propofol is particularly intriguing because of its rapid onset of action and established clinical use; however, the cellular, molecular, and circuit mechanisms through which propofol influences fear memory reconsolidation remain largely unknown. Moreover, the sedative and anesthetic properties of propofol may constrain its direct therapeutic application for fear‐related disorders. Therefore, elucidating the cell type‐specific and circuit mechanisms by which propofol modulates fear memory reconsolidation may not only clarify how propofol‐sensitive neural pathways regulate maladaptive memory, but also identify downstream molecular and circuit targets that could reproduce its beneficial effects without inducing general anesthesia.

In this study, we combined in vivo fiber photometry, pharmacological approaches, chemogenetic manipulations, and behavioral paradigms to demonstrate that propofol attenuates subsequent fear memory expression by acting during the reconsolidation window. Mechanistically, propofol suppresses the activity of D2 receptor‐expressing neurons in the medial prefrontal cortex (mPFC) and reduces reconsolidation‐associated transmission through their projection to the basolateral amygdala (BLA). Chemogenetic activation of the mPFC^D2^‐BLA pathway counteracts the effect of propofol, whereas selective disruption of D2 receptor signaling in BLA‐projecting mPFC neurons diminishes its efficacy, supporting a critical role for D2 receptor‐dependent regulation of this projection. Building on these findings, we further demonstrate that pharmacological activation of D2 receptors within the mPFC recapitulates the fear‐attenuating effect of propofol, suggesting prefrontal D2 receptor signaling may provide a pharmacological strategy for modulating maladaptive fear memories without the need for general anesthesia.

## 2 Results

### 2.1 Propofol Attenuates Subsequent Fear Memory Expression by Modulating Fear Memory Reconsolidation

To establish the behavioral paradigm for subsequent experiments, we first performed fear conditioning, fear memory retrieval, and fear memory recall. Mice received five CS‐foot shock pairings in Context A to establish fear memory. One day later, a single 20‐s CS was presented in Context B to retrieve the established fear memory and initiate reconsolidation. Subsequent fear memory expression was assessed 24 h later by presenting four CSs in the same context. Given the potential of general anesthetics to modulate fear memory reconsolidation, we systematically evaluated the effects of different anesthetic agents using a cued Pavlovian fear conditioning paradigm in mice (Figure 1A). Fear conditioning produced robust freezing responses, and freezing levels during memory retrieval were comparable across treatment groups. Notably, mice administered propofol intraperitoneally after memory retrieval showed significantly lower freezing levels during the subsequent memory recall test than mice treated with saline, midazolam, etomidate, or dexmedetomidine (Figure 1B). These results suggest that propofol selectively reduces subsequent fear memory expression when administered within the reconsolidation window.

**FIGURE 1:**
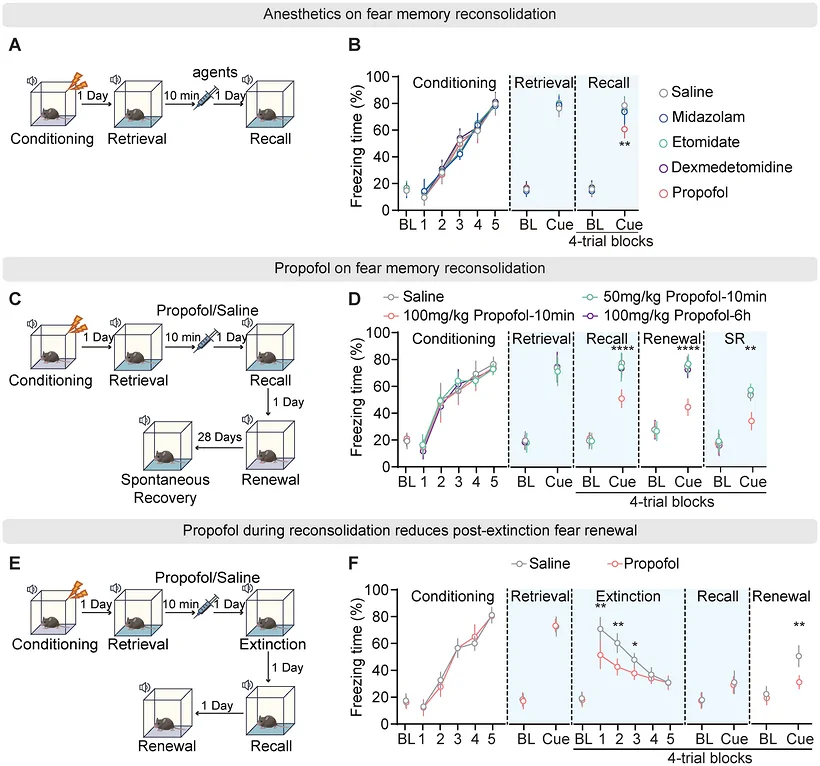
Propofol attenuates subsequent fear memory expression by modulating fear memory reconsolidation. (A) Schematic diagram of the behavioral experimental protocol. (B) Freezing responses to the CS in saline‐, midazolam‐, etomidate‐, dexmedetomidine‐, and propofol‐treated mice during fear conditioning, retrieval, and recall. Freezing responses were calculated for individual trials. Statistics were as follows: one‐way ANOVA followed by Tukey's test; retrieval test, p = 0.6842; recall test, p = 0.0013 (n = 8 mice per group). (C) Schematic diagram of behavioral experimental protocol. (D) Freezing responses to the context alone (baseline) or to the CS in saline‐ and propofol‐treated mice. Freezing responses during conditioning and retrieval were calculated as the percentage of time spent freezing during individual CS presentations, whereas those during recall, renewal, and spontaneous recovery were calculated as the percentage of time spent freezing during CS presentation. The same convention was applied to all behavioral analyses. Statistics are as follows: one‐way ANOVA followed by Tukey's multiple‐comparisons test; retrieval test, p = 0.7196; Recall test, *p* < 0.0001. Renewal test, *p* < 0.0001. spontaneous recovery test, p = 0.0023 (n = 8–12 mice per group). (E) Schematic diagram of behavioral experimental protocol. (F) Freezing responses to the context alone (baseline) or to the CS in saline‐ and propofol‐treated mice undergoing extinction training. Freezing responses during conditioning and retrieval were calculated for individual CS presentations, whereas responses during extinction, recall, and renewal were calculated as the mean freezing response across four consecutive trials. The same convention was applied throughout the behavioral analyses. Statistics were as follows: two‐way ANOVA followed by Tukey's multiple‐comparisons test; retrieval test, p = 0.5473. extinction trials 17–20, p = 0.3475 recall test, p = 0.6214. renewal test, p = 0.0084 (n = 8–12 mice per group). Statistical significance was determined by one‐way or two‐way ANOVA followed by Tukey's test. Data are presented as the mean ± SEM. ^*^ *p* < 0.05; ^**^ *p* < 0.01; ^****^ *p* < 0.0001.

To further characterize the temporal specificity and dose dependence of this effect, as well as its impact on subsequent return of fear, we examined the effects of propofol administration during the reconsolidation window on fear memory recall, renewal, and spontaneous recovery (SR) (Figure 1C). Retrieved memories remain transiently labile before undergoing reconsolidation, providing a limited temporal window during which the memory trace can be modified. Previous studies indicate that this reconsolidation window largely closes within approximately 6 h after retrieval [10]. We therefore compared propofol administration at 10 min vs. 6 h after fear memory retrieval, representing time points within and outside the expected reconsolidation window, respectively. Mice receiving 100 mg/kg propofol 10 min after retrieval exhibited significantly lower freezing than saline‐treated mice during subsequent recall, renewal, and spontaneous recovery, whereas propofol administered 6 h after retrieval had no significant effect. Moreover, 50 mg/kg propofol administered 10 min after retrieval failed to alter subsequent freezing compared with saline treatment (Figure 1D). These findings demonstrate that the fear‐attenuating effect of propofol is dose‐dependent and temporally restricted to the reconsolidation window.

We next examined whether propofol affected other stages of fear memory processing. Administration of propofol during either fear memory consolidation or extinction produced no significant effect on subsequent fear responses (Figure S1A–D). To determine whether memory retrieval was required for the effect of propofol, we administered propofol or saline in the absence of prior memory retrieval. Under this condition, propofol did not alter subsequent fear memory expression (Figure S1E,F). Together, these experiments indicate that the effect of propofol is retrieval‐dependent and selective for the reconsolidation process, rather than reflecting a general disruption of fear memory consolidation or extinction.

We further asked whether propofol treatment during reconsolidation could reduce the return of fear following subsequent extinction training. Mice received propofol or saline after memory retrieval and underwent extinction training the following day (Figure 1E). Although both groups received identical extinction training, mice treated with propofol during reconsolidation exhibited significantly lower freezing during the subsequent renewal test than saline‐treated controls (Figure 1F). Thus, propofol administration during reconsolidation reduces post‐extinction fear renewal, suggesting that its effect persists even after subsequent extinction learning.

To exclude potential nonspecific cognitive effects, we assessed whether propofol affected working memory using the Y‐maze test. Propofol administered at the tested time points did not significantly alter spontaneous alternation performance (Figure S1I). We also performed the open field test on the day of memory recall to assess locomotor activity and anxiety‐like behavior. Propofol had no significant effect on either locomotor activity or anxiety‐like behavior (Figure S1G,H). Together, these findings demonstrate that propofol administered within the reconsolidation window selectively reduces subsequent fear memory expression and multiple forms of fear recurrence, without detectable impairment of working memory or changes in locomotor and anxiety‐like behavior.

### 2.2 Propofol Inhibits mPFC D2 Neurons to Modulate Fear Memory Reconsolidation

Given the effects of propofol on fear memory, we next sought to identify the brain regions involved in fear memory reconsolidation and responsive to propofol treatment. We employed c‐Fos staining to screen brain regions activated during fear memory reconsolidation and sensitive to propofol (Figure S2A). Fear‐conditioned mice underwent the indicated experimental procedures and were perfused 90 min after treatment. C‐Fos staining revealed that the density of c‐Fos‐positive cells in the BLA and mPFC was significantly increased during fear memory reconsolidation compared with control conditions. In contrast, no significant changes were observed in the nucleus accumbens (NAc), paraventricular nucleus of the thalamus (PVT), suprachiasmatic nucleus (SCN), central amygdala (CeA), dentate gyrus (DG), ventral tegmental area (VTA), periaqueductal gray (PAG), dorsal raphe nucleus (DRN), or locus coeruleus (LC). Notably, propofol significantly reduced c‐Fos‐positive cell density in the mPFC but not in the BLA (Figure S2B,C). These results suggest that the mPFC is recruited during fear memory reconsolidation and represents a propofol‐sensitive brain region, consistent with previous findings implicating mPFC glutamatergic neurons in fear memory processing [18].

To directly monitor mPFC neuronal activity, we next performed fiber photometry by expressing AAV‐CaMKII‐GCaMP6m in the mPFC. CaMKII^+^ neuronal activity increased markedly in response to a single 20 s retrieval cue, whereas systemic administration of propofol (100 mg/kg, i.p.) significantly suppressed this activity (Figure S3A–C). To determine whether inhibition of mPFC excitatory neurons contributes to the behavioral effect of propofol, we chemogenetically activated mPFC CaMKII^+^ neurons. Chemogenetic activation of mPFC CaMKII^+^ neurons counteracted the propofol‐induced reduction in subsequent fear memory expression (Figure S3F,G). Chemogenetic inhibition of CaMKII^+^ neurons mimicked the fear‐attenuating effect of propofol (Figure S3D,E). Together, these results indicate that suppression of mPFC excitatory neuronal activity is functionally required for the effect of propofol on fear memory reconsolidation.

Previous studies have identified molecularly and functionally distinct populations of mPFC glutamatergic neurons expressing dopamine D1 or D2 receptors [20]. However, their respective roles in fear memory reconsolidation remain poorly understood. We therefore first quantified the proportion of c‐Fos^+^ cells that co‐expressed mCherry (D2^+^) in the mPFC across control, cue‐exposed (fear memory retrieval), and propofol‐treated groups. Cue exposure increased the proportion of c‐Fos^+^/mCherry^+^ double‐positive cells among total c‐Fos^+^ cells, whereas propofol attenuated this increase (Figure S4A,B). We next used in vivo fiber photometry to compare D1‐ and D2‐expressing neuronal activity during fear memory reconsolidation (Figure 2A). mPFC D2 neurons showed robust retrieval‐associated activation, whereas D1 neurons did not exhibit a comparable response (Figure 2B,C), suggesting a preferential involvement of D2 neurons in fear memory reconsolidation.

**FIGURE 2:**
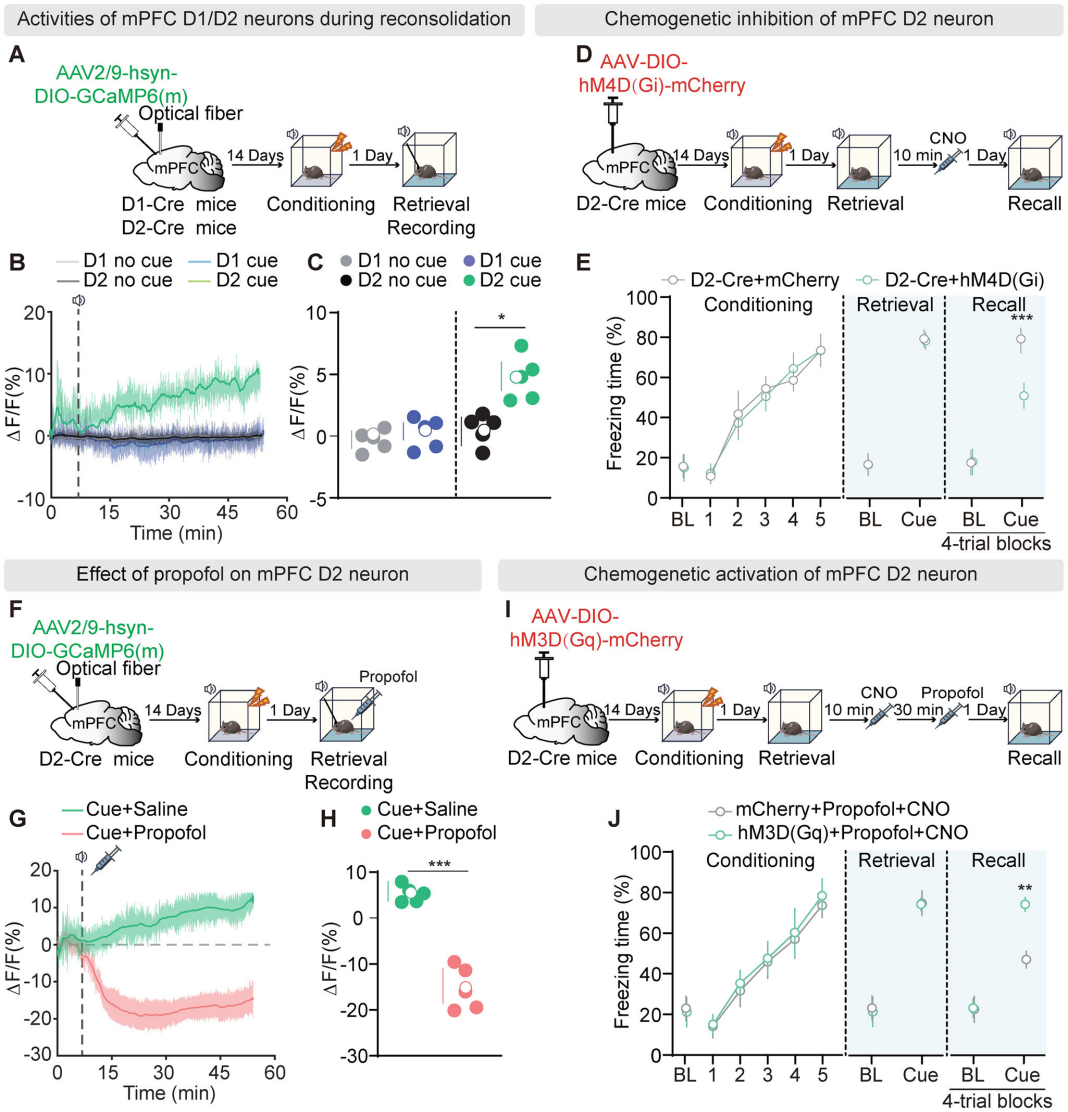
Propofol inhibits mPFC D2 neurons to modulate fear memory reconsolidation. (A) Schematic of fiber implantation over the mPFC following expression of GCaMP6m in D1‐ or D2 receptor‐expressing neurons of D1‐Cre or D2‐Cre mice, respectively. (B) GCaMP6m signals from mPFC D1 or D2 neurons aligned to the onset of no‐CS or CS presentation. (C) Quantification of changes in GCaMP6m fluorescence signals following no‐CS or CS presentation (n = 5 mice per group). (D) Schematic diagram of the behavioral experimental protocol. (E) Freezing responses to the context alone (baseline) or to the CS in D2‐Cre mice expressing mCherry or hM4D(Gi) in mPFC D2 neurons. Statistics were as follows: unpaired Student's *t*‐test; retrieval test, p = 0.7351; recall test, p = 0.0004 (n = 8 mice per group). (F) Schematic of fiber implantation over the mPFC following expression of GCaMP6m in D2 receptor‐expressing neurons of D2‐Cre mice. (G) GCaMP6m signals from mPFC D2 neurons aligned to the onset of CS presentation in saline‐ or propofol‐treated mice (100 mg/kg). (H) Quantification of changes in GCaMP6m signals following CS presentation in saline‐ or propofol‐treated mice (100 mg/kg) (n = 5 mice per group). (I) Schematic diagram of the behavioral experimental protocol. (J) Freezing responses to the context alone (baseline) or to the CS in D2‐Cre mice expressing mCherry or hM3D(Gq) during propofol treatment. Statistics were as follows: unpaired Student's *t*‐test; retrieval test, p = 0.8634; recall test, p = 0.0028. (n = 8 mice per group). Statistical significance was determined by unpaired Student's *t*‐test and one‐way ANOVA followed by Tukey's multiple‐comparisons test, as appropriate. Data are presented as the mean ± SEM. ^*^ *p* < 0.05; ^**^ *p* < 0.01; ^***^ *p* < 0.001.

To test whether mPFC D2 neuronal activity is required for fear memory reconsolidation, we injected rAAV‐DIO‐hM4D(Gi)‐mCherry or rAAV‐DIO‐mCherry into the mPFC of D2‐Cre mice (Figure 2D). Chemogenetic inhibition of mPFC D2 neurons during reconsolidation significantly reduced subsequent fear memory expression (Figure 2E), supporting a causal role for this neuronal population. In contrast, chemogenetic activation of mPFC D1 neurons did not significantly influence the effect of propofol (Figure S4G,H). To determine whether the effects of D1 and D2 neuronal inhibition depended on memory retrieval, we repeated these chemogenetic manipulations in the absence of memory retrieval (Figure S4C,E). Under these conditions, inhibition of either population had no significant effect on subsequent fear memory expression (Figure S4D,F). These findings indicate that the contribution of mPFC D2 neurons is retrieval‐dependent and linked specifically to the reconsolidation process.

Given the selective involvement of mPFC D2 neurons in fear memory reconsolidation, we next asked whether this neuronal population mediates the effect of propofol. In vivo fiber photometry showed that propofol markedly suppressed the retrieval‐associated activation of mPFC D2 neurons during the reconsolidation window (Figure 2F–H). To test whether this inhibition was required for the behavioral effect of propofol, we chemogenetically activated mPFC D2 neurons during propofol treatment (Figure 2I). Sustained activation of mPFC D2 neurons abolished the propofol‐induced reduction in subsequent fear memory expression (Figure 2J). These results demonstrate that inhibition of mPFC D2 neurons is a critical cellular mechanism underlying the effect of propofol on fear memory reconsolidation.

Collectively, these findings reveal distinct roles for mPFC D1‐ and D2 receptor‐expressing neurons during fear memory reconsolidation and identify D2 neurons as the principal propofol‐responsive population. By suppressing the retrieval‐associated activation of mPFC D2 neurons, propofol reduces subsequent fear memory expression, highlighting this neuronal population as a potential target for modulating maladaptive fear memories.

### 2.3 mPFC D2 Receptor Activation Mediates the Effect of Propofol on Fear Memory Reconsolidation

We next sought to identify the neurotransmitter mechanism through which propofol suppresses mPFC D2 neuronal activity during fear memory reconsolidation. Given the established role of the noradrenergic (NE) system, particularly β‐adrenoceptors, in fear memory reconsolidation [21, 22, 23], we next examined whether NE signaling contributes to the effect of propofol. Using fiber photometry, we found that administration of 100 mg/kg propofol significantly increased NE levels in the mPFC (Figure S5A–C). However, ablation of locus coeruleus (LC) neurons by injection of AAV‐hSyn‐DTA did not abolish the effect of propofol on fear memory reconsolidation (Figure 3A,B). Similarly, pharmacological blockade of β‐adrenoceptors in the mPFC failed to prevent the effect of propofol on reconsolidation (Figure 3C,D). Together, these findings indicate that although propofol elevates mPFC NE levels, mPFC β‐adrenergic signaling was not required for its effect on fear memory reconsolidation.

**FIGURE 3:**
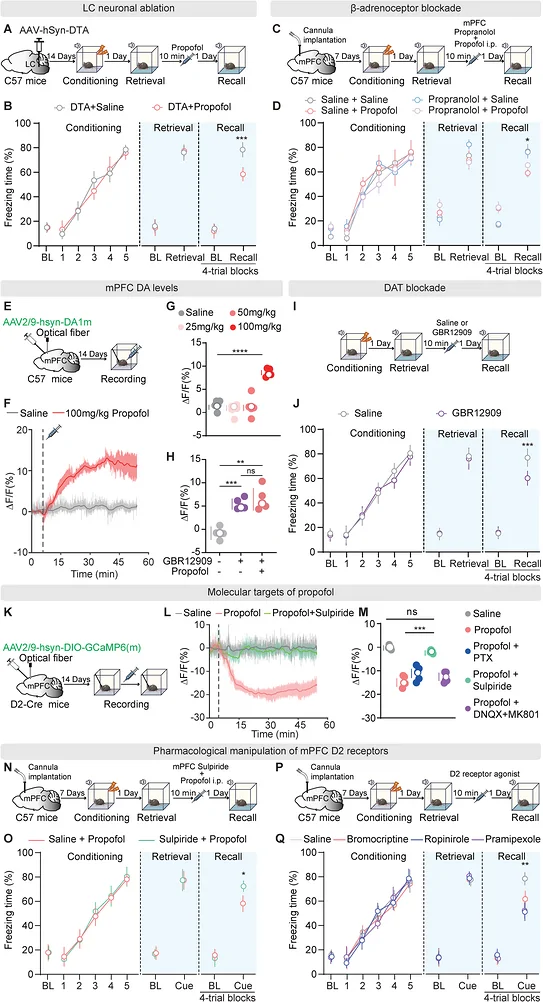
mPFC D2 receptor activation mediates the effect of propofol on fear memory reconsolidation. (A) Schematic diagram of the behavioral experimental protocol. (B) Freezing responses to the context alone (baseline) or to the CS in AAV‐hSyn‐DTA‐expressing mice. Statistics are as follows: unpaired Student's *t*‐test; retrieval test, p = 0.8631; recall test, p = 0.0003 (n = 8 mice per group). (C) Schematic diagram of intra‐mPFC infusion of the β‐adrenergic receptor antagonist propranolol combined with systemic propofol administration (i.p.), and the behavioral experimental protocol. (D) Freezing responses to the CS in mice receiving intra‐mPFC saline or propranolol together with systemic saline or propofol (i.p.). The four treatment groups were saline + saline, propranolol + saline, saline + propofol, and propranolol + propofol. Statistics were as follows: one‐way ANOVA followed by Tukey's multiple‐comparisons test; retrieval test, p = 0.7246; recall test, p = 0.0372 (n = 8–12 mice per group). (E) Schematic of fiber implantation over the mPFC following expression of the dopamine sensor DA1m in C57BL/6J mice. (F) DA sensor signals from the mPFC aligned to the time of saline or propofol administration (100 mg/kg). (G) Quantification of changes in DA sensor signals after administration of saline or different doses of propofol (25, 50, 100 mg/kg) in the mPFC of C57BL/6J mice (n = 5 mice per group). (H) Quantification of changes in DA‐1m signals after administration of saline + saline, saline + GBR12909, and GBR12909 + propofol (100 mg/kg) (n = 5 mice per group). (I) Schematic diagram of the behavioral experimental protocol. (J) Freezing responses to the CS in saline‐ and GBR12909‐treated mice during fear conditioning, retrieval, and recall. Statistics were as follows: unpaired t‐test; retrieval test, p = 0.6118; recall test, p = 0.0008 (n = 8 mice per group). (K) Schematic of fiber implantation over the mPFC following expression of GCaMP6m in D2 receptor‐expressing neurons of D2‐Cre mice. (L) GCaMP6m signals from mPFC D2 neurons aligned to the time of the administration of saline, propofol, and propofol+ sulpiride, or other indicated pharmacological treatments. (M) Quantification of changes in GCaMP6m signals after administration of saline, propofol + saline, propofol + PTX, propofol + MK801 + DNQX, and propofol + sulpiride in mPFC D2 neurons of D2‐Cre mice (n = 5 mice per group). (N) Schematic diagram of the intra‐mPFC infusion of the D2 receptor antagonist sulpiride and the behavioral experimental protocol. (O) Freezing responses to the context alone (baseline) or to the CS in mice treated with propofol plus intra‐mPFC saline or propofol plus intra‐mPFC sulpiride. Statistics are as follows: unpaired Student’s t‐test; retrieval test, p = 0.8846; recall test, p = 0.0216 (n = 8–12 mice per group). (P) Schematic diagram of the intra‐mPFC infusion of the D2 receptor agonists bromocriptine, ropinirole, or pramipexole and the behavioral experimental protocol. (Q) Freezing responses to the context alone (baseline) or to the CS in mice receiving intra‐mPFC saline or D2 receptor agonists. Statistics were as follows: one‐way ANOVA followed by Tukey's multiple‐comparisons test; retrieval test, p = 0.9826; recall test, p = 0.0056 (n = 8‐ 12 mice per group). Statistical significance was determined by one‐way ANOVA followed by Tukey's multiple comparisons test and unpaired Student's *t*‐test, as appropriate. Data are presented as the mean ± SEM. ^*^ *p* < 0.05; ^**^ *p* < 0.01; ^***^ *p* < 0.001; ^****^ *p* < 0.0001.

Given our previous finding that propofol increases dopamine levels in the NAc [24], together with the established involvement of dopamine in memory reconsolidation [25], we next examined whether dopaminergic signaling contributes to the effect of propofol in the mPFC. Using a dopamine sensor, we found that propofol significantly increased extracellular dopamine levels in the mPFC (Figure 3E–G). To determine whether dopamine transporter (DAT)‐dependent reuptake contributes to this increase, we examined the effects of propofol after pretreatment with the DAT blocker GBR12909. GBR12909 itself markedly elevated mPFC dopamine levels and occluded a further dopamine increase following propofol administration (Figure 3H). This pharmacological occlusion is consistent with a contribution of DAT‐dependent dopamine reuptake to the propofol‐induced elevation of extracellular dopamine. We next asked whether increasing extracellular dopamine through DAT inhibition could mimic the behavioral effect of propofol during reconsolidation (Figure 3I). Mice receiving systemic GBR12909 during the reconsolidation phase exhibited significantly lower freezing during subsequent memory recall than saline‐treated mice (Figure 3J). Together, these findings implicate increased mPFC dopaminergic signaling in the modulation of fear memory reconsolidation by propofol and support the involvement of DAT‐dependent dopamine regulation.

We next investigated whether elevated dopamine suppresses mPFC D2 neuronal activity through D2 receptor signaling. Because propofol is known to potentiate GABA_A_ receptor‐mediated inhibition and may suppress excitatory glutamatergic signaling [26], its inhibitory effect on D2 neurons could theoretically arise through several mechanisms. We therefore considered three possibilities: (1) D2 receptor activation secondary to increased extracellular dopamine; (2) potentiation of GABA_A_ receptor‐mediated inhibition; or (3) suppression of excitatory glutamatergic transmission. To distinguish among these mechanisms, we expressed GCaMP6m in the mPFC of D2‐Cre mice using AAV‐hSyn‐DIO‐GCaMP6m and conducted in vivo fiber photometry (Figure 3K). The propofol‐induced suppression of D2 neuronal activity was markedly attenuated by the D2 receptor antagonist sulpiride, whereas blockade of GABA_A_, AMPA, or NMDA receptors with PTX, DNQX, or MK801, respectively, did not significantly alter this effect (Figure 3L,M). These results support a mechanism in which propofol suppresses mPFC D2 neuronal activity predominantly through dopamine‐dependent activation of D2 receptors rather than through direct modulation of GABA_A_ receptor‐ or glutamatergic receptor‐dependent mechanisms under these experimental conditions.

To establish the functional role of D2 receptor signaling in fear memory reconsolidation, we next performed local pharmacological manipulations of D2 receptors in the mPFC. Intra‐mPFC infusion of the D2 receptor antagonist sulpiride during systemic propofol treatment attenuated the propofol‐induced reduction in subsequent fear memory expression (Figures 3N,O). Conversely, local administration of the D2 receptor agonists ropinirole, pramipexole, or bromocriptine into the mPFC immediately after memory retrieval significantly reduced freezing during subsequent memory recall (Figure 3P,Q). Thus, local D2 receptor activation was sufficient to recapitulate the fear‐attenuating effect of propofol during the reconsolidation window.

To further evaluate the potential translational relevance of pharmacological D2 receptor activation, we assessed the effect of systemic bromocriptine administration during fear memory reconsolidation (Figure S6A). Mice receiving bromocriptine during the reconsolidation phase exhibited significantly lower freezing during subsequent memory recall and renewal than saline‐treated mice (Figure S6B). Moreover, bromocriptine did not significantly affect anxiety‐like behavior, working memory, or locomotor activity under these experimental conditions (Figure S6C–E). These findings indicate that pharmacological activation of D2 receptors can reproduce the fear‐attenuating effect of propofol without detectable effects on working memory or locomotor activity.

Together, these findings support a model in which propofol increases extracellular dopamine in the mPFC, engages D2 receptor signaling, and thereby suppresses the activity of mPFC D2 neurons during the reconsolidation window. Pharmacological activation of D2 receptors recapitulates this effect and reduces subsequent fear memory expression, identifying mPFC D2 receptor signaling as a key molecular mechanism underlying the effect of propofol on fear memory reconsolidation.

### 2.4 Propofol Suppresses mPFC^D2^‐BLA Circuit to Modulate Fear Memory Reconsolidation

To identify the downstream circuitry through which mPFC D2 neurons regulate fear memory reconsolidation, we employed an AAV‐based tracing approach to map the projection targets of mPFC D2 neurons (Figure 4A). Among the regions examined, the BLA exhibited the most prominent innervation from mPFC D2 neurons (Figure 4B,C). Given that the established role of the BLA in fear memory reconsolidation [27, 28, 29]and the prominent mPFC D2 input to the BLA observed here, we next examined whether synaptic transmission from mPFC D2 neurons to the BLA is dynamically altered during reconsolidation and whether this transmission is modulated by propofol.

**FIGURE 4:**
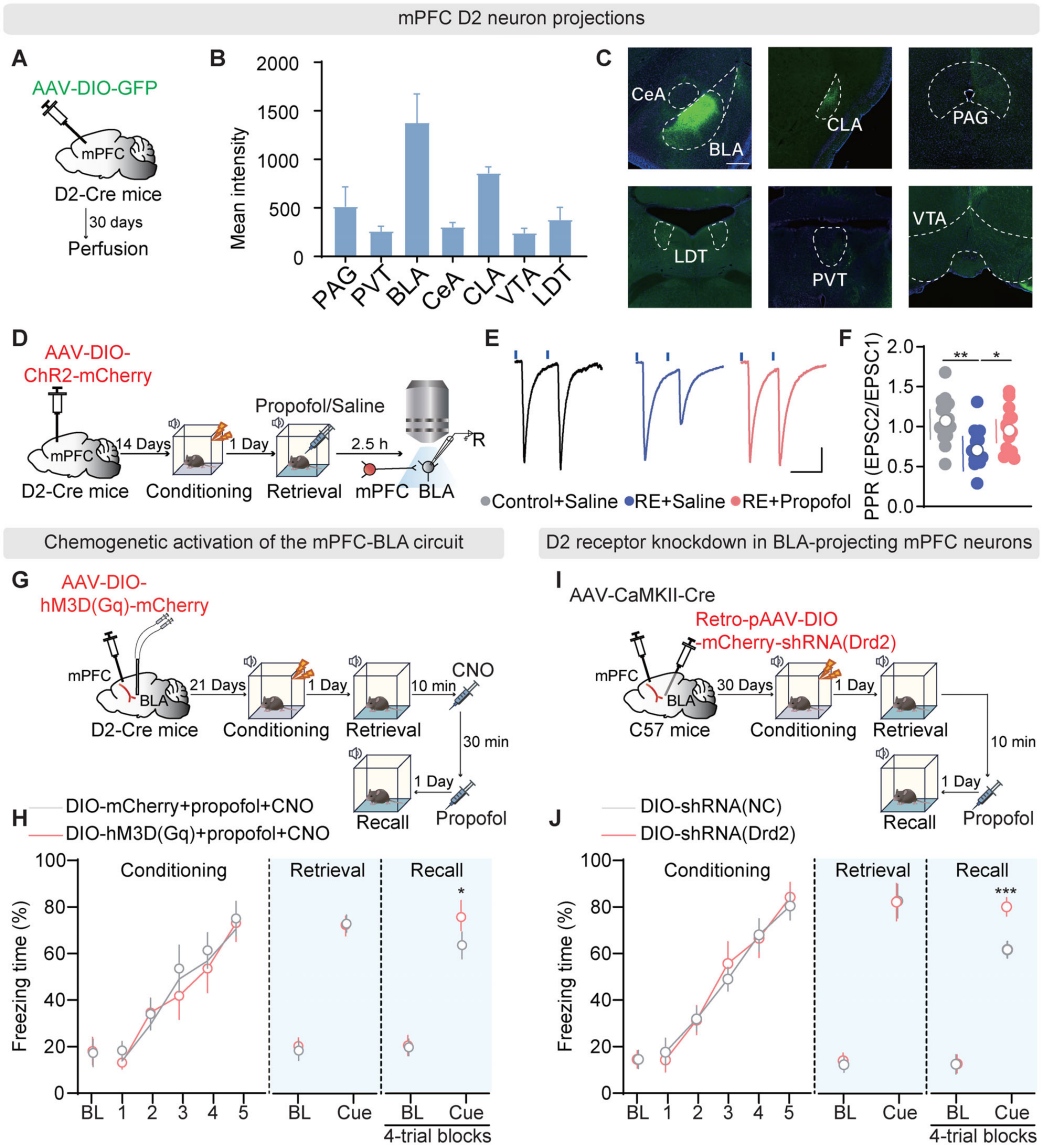
Propofol inhibits Reconsolidation‐Associated Enhancement of mPFC^D2^‐BLA transmission to modulate fear memory reconsolidation. (A) Schematic of anterograde tracing of mPFC D2 neurons following AAV‐DIO‐GFP injection into the mPFC of D2‐Cre mice. (B) Quantification of GFP fluorescence in brain regions that receive projections from mPFC D2 neurons. (C) Representative GFP fluorescence in brain regions receiving projections from mPFC D2 neurons. Scale bar, 400 µm. (D) Schematic diagram of ex vivo electrophysiological recording protocol. (E) Representative paired‐pulse EPSC traces from BLA neurons following optogenetic stimulation of mPFC D2 neuronal terminals in control, reconsolidation (RE), and propofol‐treated mice. Scale bars: 50 ms, 50 pA. (F) Quantification of the paired‐pulse ratio (PPR) of optogenetically evoked EPSCs in BLA neurons from control, reconsolidation, and propofol‐treated mice (n = 15 cells from 3 mice per group). (G) Schematic diagram of the behavioral experimental protocol. (H) Freezing responses to the context alone (baseline) or to the CS in D2‐Cre mice expressing mCherry or hM3D(Gq) in mPFC D2 neurons, with local CNO infusion into the BLA to selectively activate mPFC D2 neuronal terminals during propofol treatment. Statistics were as follows: unpaired Student's *t*‐test; retrieval test, p = 0.7035; recall test, p = 0.0295(n = 8 mice per group). (I) Schematic diagram of the behavioral experimental protocol. (J) Freezing responses to the context alone (baseline) or to the CS in propofol‐treated mice with projection‐specific Drd2 knockdown or control shRNA. Statistics were as follows: unpaired Student's *t*‐test; retrieval test, p = 0.9355; recall test, p = 0.0004 (n = 8 mice per group). Statistical significance was determined by one‐way ANOVA followed by Tukey's multiple comparisons test and unpaired Student's t‐test, as appropriate. Data are presented as the mean ± SEM. ^*^ *p* < 0.05; ^**^ *p* < 0.01 ；***p < 0.001.

To assess functional transmission within this pathway, we injected AAV‐DIO‐ChR2‐mCherry into the mPFC of D2‐Cre mice and optogenetically stimulated mPFC D2 neuronal terminals in the BLA during ex vivo whole‐cell recordings (Figure 4D). Fear memory reconsolidation was associated with a significant decrease in the paired‐pulse ratio (PPR) of optogenetically evoked responses in BLA neurons, consistent with an increase in presynaptic release probability. Notably, propofol treatment significantly increased the PPR toward control levels, indicating a reversal of the reconsolidation‐associated enhancement in presynaptic transmission (Figure 4E,F). These findings suggest that fear memory reconsolidation enhances presynaptic transmission from mPFC D2 neurons to the BLA, whereas propofol suppresses this reconsolidation‐associated enhancement.

To determine whether activity of the mPFC^D2^‐BLA circuit contributes to the behavioral effect of propofol, we expressed AAV‐DIO‐hM3D (Gq) in mPFC D2 neurons of D2‐Cre mice and then implanted a guide cannula into the BLA for local CNO administration (Figure 4G). After 3 weeks of viral expression, CNO was infused into the BLA to selectively activate hM3D(Gq)‐expressing terminals of mPFC D2 neurons during the reconsolidation period, followed by systemic propofol administration. Chemogenetic activation of the mPFC^D2^‐BLA projection abolished the propofol‐induced reduction in subsequent fear memory expression (Figure 4H), supporting the functional contribution of suppression of this projection to the behavioral effect of propofol.

We next asked whether D2 receptor signaling in BLA‐projecting mPFC neurons is required for the effect of propofol. To selectively reduce D2 receptor expression in this projection‐defined neuronal population, we injected AAV‐CaMKII‐Cre into the mPFC and a retrogradely transported Cre‐dependent vector carrying either Drd2‐targeting shRNA [Retro‐AAV‐CBG‐DIO‐mCherry‐miR30shRNA(Drd2) or Retro‐AAV‐CBG ‐ DIO‐mCherry‐miR30shRNA (NC)] into the BLA. This intersectional strategy enabled preferential Drd2 knockdown in excitatory mPFC neurons projecting to the BLA. After 30 days of viral expression, reduced D2 receptor expression was verified in the mPFC (Figure S7A,B), followed by behavioral tests (Figure 4I). Projection‐specific Drd2 knockdown significantly attenuated the effect of propofol on subsequent fear memory expression (Figure 4J), indicating that D2 receptor signaling in BLA‐projecting mPFC neurons contributes critically to the effect of propofol on fear memory reconsolidation.

Together, these findings identify the mPFC^D2^‐BLA pathway as a functionally relevant circuit substrate for fear memory reconsolidation and demonstrate that propofol suppresses reconsolidation‐associated enhancement of this projection through a mechanism that depends, at least in part, on D2 receptor signaling in BLA‐projecting mPFC neurons.

## 3 Discussion

The present study identifies mPFC D2 receptor‐expressing neurons as a key cellular substrate regulating fear memory reconsolidation and reveals a dopamine‐dependent mechanism involving D2 receptor signaling through which propofol modulates this process. Our findings further implicate the mPFC^D2^‐BLA pathway as a critical downstream circuit contributing to the effect of propofol, with D2 receptor signaling in BLA‐projecting mPFC neurons playing an important role. Together, these results define a cell type‐ and circuit‐specific framework for understanding how propofol modifies maladaptive fear memories and identify prefrontal D2 receptor signaling as a potential pharmacological target for modulating memory reconsolidation.

Accumulating preclinical and clinical evidence indicates that propofol can influence multiple forms of memory, including fear‐related memories [30, 31, 32, 33]. These observations have raised interest in whether anesthetic‐sensitive mechanisms could be exploited to modify maladaptive memories. In particular, a recent clinical study reported that propofol interfered with the reconsolidation of emotionally negative memories, supporting the possibility that propofol‐sensitive pathways may be relevant to reconsolidation‐based interventions [17]. Reconsolidation provides a transient period after retrieval during which established memories become labile and susceptible to modification [34, 35]. In the present study, propofol administered within this reconsolidation window reduced subsequent fear memory expression, renewal, and spontaneous recovery, whereas administration outside the reconsolidation window or in the absence of retrieval was ineffective. These temporal and retrieval‐dependent effects argue against a nonspecific suppression of fear expression and instead support a reconsolidation‐dependent mechanism. Moreover, propofol administered during reconsolidation remained compatible with subsequent extinction training and reduced later fear renewal. These findings suggest that propofol‐sensitive mechanisms could potentially be combined with behavioral interventions to promote more persistent attenuation of maladaptive fear memories.

The pivotal role of the mPFC in fear memory reconsolidation has been demonstrated by several studies [36, 37]. Moreover, preliminary evidence suggests that inhibiting prefrontal neuronal activity during the reconsolidation window can attenuate subsequent fear memory expression [38, 39]. However, the specific cell types, circuits, and molecular mechanisms underlying these effects remain incompletely understood. The current study revealed that mPFC D2 receptor‐expressing neurons show prominent retrieval‐associated activation and a functional contribution to fear memory reconsolidation, whereas D1 receptor‐expressing neurons do not exhibit a comparable response. Furthermore, our findings demonstrate that fear memory reconsolidation is associated with enhanced synaptic transmission in the mPFC^D2^‐BLA pathway and that propofol suppresses this reconsolidation‐associated enhancement. Chemogenetic activation of this projection counteracted the behavioral effect of propofol, further supporting its functional involvement. These findings reveal functional heterogeneity among prefrontal dopamine receptor‐defined neuronal populations and identify mPFC D2 neurons and their projection to the BLA as a cellular and circuit substrate through which propofol modulates fear memory reconsolidation.

The noradrenergic system has long been implicated in fear memory reconsolidation [23, 40]. Notably, the present study indicated that propofol modulated fear memory reconsolidation largely independently of β‐adrenergic signaling, despite increasing NE levels in the mPFC. In addition to the noradrenergic system, emerging evidence also points to a modulatory role for the dopaminergic system in several fear‐related processes. Nevertheless, whether targeted modulation of dopaminergic signaling, for instance, via selective agonists or antagonists, can effectively modulate fear memory reconsolidation and thereby exert therapeutic effects has remained unclear. Here, propofol increased extracellular dopamine in the mPFC and reduced subsequent fear memory expression when administered during the reconsolidation window. Importantly, local administration of several D2 receptor agonists, including bromocriptine, pramipexole, and ropinirole, recapitulated this fear‐attenuating effect. Notably, our present results showed that 100 mg/kg, but not 50 mg/kg, propofol attenuated fear memory reconsolidation, a dose dependence that may be related to differences in the magnitude of dopamine elevation. In our previous study, in vivo PET‐CT imaging indicated that 100 mg/kg, but not 50 mg/kg, propofol reduced DAT availability and increased dopamine levels in the mouse brain [24]. Together with the present pharmacological occlusion experiments using the DAT inhibitor GBR12909, these observations are consistent with a contribution of DAT‐dependent dopamine regulation to the effect of propofol. Furthermore, we identified the D2 receptor in the mPFC as a key molecular target through which dopaminergic signaling contributes to these effects.

These findings raise the possibility of developing non‐anesthetic pharmacological strategies targeting D2 receptor signaling to modulate pathological fear memories. In addition, previous work has shown that elevated dopamine acting on D2 receptors impairs the induction of long‐term potentiation (LTP) in the mPFC [41]. Such D2 receptor‐dependent regulation of synaptic plasticity may provide one mechanism through which increased dopamine alters the activity and plasticity of mPFC D2 neurons during reconsolidation. Together, our work links prefrontal dopaminergic signaling to a defined neuronal population and projection pathway involved in fear memory reconsolidation, providing a mechanistic foundation for future studies of reconsolidation‐based interventions.

Beyond the mechanistic insights, our findings suggest potential translational relevance. The identification of mPFC D2 receptors as a key node in fear memory reconsolidation offers a pharmacologically accessible molecular target. Notably, the D2 receptor agonists we tested ‐ bromocriptine, pramipexole, and ropinirole ‐ are already clinically approved for other indications, which may facilitate future evaluation of whether D2 receptor modulation can influence maladaptive memory reconsolidation in humans [42, 43, 44]. However, these agents act broadly on dopaminergic systems and can produce clinically relevant adverse effects; therefore, their efficacy, dosing, temporal relationship to memory retrieval, and safety would require careful investigation before application to fear‐related disorders. Their use as adjuncts to behavioral interventions targeting the reconsolidation window therefore remains an important question for future clinical studies. Furthermore, our finding that mPFC D2 neurons contribute to fear memory reconsolidation identifies this neuronal population as a mechanistic substrate that may help guide circuit‐level neuromodulation strategies targeting the mPFC. Techniques such as transcranial magnetic stimulation and transcranial direct current stimulation can modulate prefrontal network activity, although they do not currently provide cell type‐specific control of D2 receptor‐expressing neurons [45, 46]. Future studies could explore whether neuromodulation of prefrontal networks or mPFC‐amygdala functional connectivity, timed to the reconsolidation window, can recapitulate the reconsolidation‐modulating effects of propofol without the need for systemic administration of pharmacological agents. Together, these considerations highlight a potential trajectory from mechanistic discovery to translational application, while emphasizing the need to determine whether the molecular and circuit mechanisms identified in mice generalize to human maladaptive fear memories.

## 4 Conclusion

Our study demonstrates that D2 receptor‐expressing neurons in the mPFC are critically involved in fear memory reconsolidation. Propofol increases prefrontal dopaminergic signaling, engages D2 receptor‐dependent inhibition of mPFC D2 neurons, and suppresses reconsolidation‐associated activity within the mPFC^D2^‐BLA pathway, thereby reducing subsequent fear memory expression. These findings identify prefrontal D2 receptor signaling and the mPFC^D2^‐BLA pathway as key molecular and circuit substrates through which propofol modulates fear memory reconsolidation and suggest potential targets for developing non‐anesthetic strategies to modify maladaptive fear memories.

## 5 Experimental Model and Subject Details

### 5.1 Animals

Male and female C57BL/6J, D1‐Cre, and D2‐Cre mice aged 8–10 weeks were used for the experiments. Both male and female mice were used for in vivo fiber photometry recordings and ex vivo electrophysiological recordings. Male mice were used for fear‐conditioning and behavioral experiments. All mice were housed 4–5 animals per cage under a 12 h light/dark cycle (lights on from 07:00 to 19:00) at 22°C–25°C, with ad libitum access to food and water. All experimental procedures were conducted in accordance with protocols approved by the Institutional Animal Care and Use Committees of ShanghaiTech University (20250507002) and Shanghai Mental Health Center (AW‐2510).

### 5.2 Method Details

#### 5.2.1 Drug Administration

For systemic drug delivery, propofol (ApexBio, C6265) was dissolved in intralipid (Sigma, I141) and further diluted with 0.9% saline. The GABA_A_ receptor antagonist picrotoxin (PTX; Tocris Bioscience), the D2 receptor antagonist sulpiride (50 mg/kg for systemical administeration and 10 µg/µL for local infusion, MedChemExpress), and clozapine‐N‐oxide (CNO;2 mg/kg, ENZO, BML‐NS105‐0025) were initially dissolved in DMSO and subsequently diluted in 0.9% saline. Bromocriptine (5 mg/kg, ApexBio), propranolol (10 mg/kg, MCE), dexmedetomidine (100 µg/kg, MCE), midazolam (10 mg/kg, Sigma‐Aldrich), etomidate (20 mg/kg, Sigma‐Aldrich), DNQX (10 mg/kg, Tocris Bioscience), and MK801(0.1 mg/kg, Tocris Bioscience), GBR12909 (10 mg/kg, ApexBio) were prepared in 0.9% saline. Systemically administered drugs were delivered intraperitoneally (i.p.) for behavioral and in vivo fiber photometry experiments. For local intracranial infusion, bromocriptine (5 µg/µL, ApexBio, M9099), pramipexole (1 µg/µL, MCE), ropinirole (1 µg/µL, MCE) was dissolved in saline.

#### 5.2.2 Stereotactic Surgery

rAAV‐hSyn‐DA1m‐WPRE‐PA, AAV2/9‐hSyn‐CaMKII‐GCaMP6m, rAAV‐hSyn‐DIO‐GCaMP6m, rAAV‐CaMKII‐hM3D(Gq)‐mCherry, rAAV‐CaMKII‐mCherry, rAAV‐DIO‐ChR2‐mCherry, rAAV‐DIO‐GFP, rAAV‐DIO‐hM4D(Gi)‐mCherry, rAAV‐DIO‐hM3D(Gq)‐mCherry, rAAV‐DIO‐mCherry, rAAV‐hSyn‐NE2.0‐WPRE‐PA, rAAV‐hSyn‐DTA, rAAV‐CaMKII‐Cre were purchased from BrainVTA (Wuhan, China). Retro‐AAV‐CBG‐DIO‐mCherry‐miR30shRNA(Drd2) and Retro‐AAV‐CBG‐DIO‐mCherry‐miR30shRNA(NC) were purchased from OBiO (Shanghai, China). Optical fibers and cannulae were purchased from RWD Life Science Co., Ltd. (Shenzhen, China). Surgical procedures were performed as previously described, with minor modifications. Briefly, anesthesia was induced with 4% isoflurane and maintained with 1.5% isoflurane. Mice were then secured in a stereotactic apparatus, and the skull was leveled before viral injection or implantation. Histological verification of viral expression and optical fiber/cannula placements is shown in Figure S8.

For fiber photometry recordings, 300 nL AAV was slowly injected into either mPFC (AP +1.6 mm, ML ±0.35 mm, DV 2.0 mm from Bregma) or BLA (AP −1.8 mm, ML ±3.25 mm, DV 4.2 mm from Bregma). Optical fibers were implanted 0.2 mm above the corresponding viral injection sites. For chemogenetic experiments, rAAV was bilaterally injected into the mPFC without optical fiber implantation. For cannula implantation, guide cannulae were stereotaxically positioned in the mPFC or BLA without viral injection, unless otherwise specified. After viral delivery, the injection needle was left in place for at least 10 min to allow viral diffusion and minimize reflux.

#### 5.2.3 Fiber Photometry

After 2 weeks of recovery and viral expression, mice underwent fiber photometry recordings. Fluorescence signals were obtained with a fiber photometry system equipped with a 488 nm excitation laser (Coherent, # OBIS LS‐488), a 505–544 nm emission filter, and a photomultiplier tube (Hamamatsu, # R3896). Analog voltage signals were digitized at 100 Hz and recorded using a Power 1401 digitizer and Spike2 software (CED, Cambridge, UK, # 1401). An optical fiber (Thorlabs, # RJPSF2) connected through a rotary joint was used to transmit light between the fiber photometry system and the implanted optical fiber while minimizing movement‐induced twisting. The laser power was adjusted to 20–40 µW to minimize photobleaching. Photometry data were exported to MATLAB R2021a for analysis. Fluorescence bleaching was corrected before calculation of normalized fluorescence changes. ΔF/F was calculated as (F‐F_0_)/F_0_, where F_0_ represented the mean baseline fluorescence during a 300 s stable pre‐treatment period. ΔF/F traces are presented as mean ± SEM. For drug‐response analyses, the mean ΔF/F value during the 60 min period after drug administration was used for statistical comparison.

#### 5.2.4 Brain Slice Electrophysiology

Adult D2‐Cre mice (8–10 weeks old) expressing DIO‐ChR2 in mPFC underwent fear conditioning and fear memory retrieval before slice preparation. Mice were anesthetized with tribromoethanol (300 mg kg^−1^, i.p.) and transcardially perfused with ice‐cold oxygenated (95% O_2_/5% CO_2_) NMDG ACSF solution, which included 93 mM NMDG, 93 mM HCl, 2.5 mM KCl, 1.25 mM NaH_2_PO_4_, 10 mM MgSO_4_·7H_2_O, 30 mM NaHCO_3_, 25 mM glucose, 20 mM HEPES, 5 mM sodium ascorbate, 3 mM sodium pyruvate, and 2 mM thiourea. Brains were rapidly dissected out and immediately transferred into an ice‐cold oxygenated NMDG ACSF solution. Coronal slices (300 µm) were prepared using a vibratome (VT1200 S, Leica). Slices containing the BLA were allowed to recover in oxygenated NMDG ACSF at 32°C for 10–15 min and subsequently transferred to standard ACSF (126 mM NaCl, 2.5 mM KCl, 1.25 mM NaH_2_PO_4_, 2 mM MgSO_4_·7H_2_O, 10 mM Glucose, 26 mM NaHCO_3_, 2 mM CaCl_2_) at room temperature for 1 h. All chemicals used in slice preparation were purchased from Sigma‐Aldrich (St. Louis, MO, USA).

During recording, slices were submerged and continuously perfused with ACSF at a rate of 3 mL/min at 28°C. Whole‐cell patch‐clamp recordings were made from BLA neurons visualized with an Olympus BX61W1 microscope equipped with GFP and mCherry filters and infrared differential interference contrast optics. Recording pipettes (3–4 MΩ) were prepared using a micropipette puller (P2000, Sutter Instrument, USA). For whole‐cell recording, the pipettes were filled with an intracellular solution containing 133 mM potassium gluconate, 18 mM NaCl, 0.6 mM EGTA, 10 mM HEPES, 2 mM Mg·ATP, and 0.3 mM Na_3_·GTP (pH 7.2, 280 mOsm). For paired pulse ratio (PPR) measurements of optogenetically evoked excitatory postsynaptic currents (EPSCs), two 2 ms light pulses separated by 50 ms were delivered to mPFC D2 neuronal terminals in the BLA every 30 s. Light intensity was adjusted to evoke EPSCs of approximately 100–200 pA. PPR was calculated as the amplitude of the second EPSC divided by that of the first EPSC (EPSC2/EPSC1).

#### 5.2.5 Behavioral Assays

Behavioral experiments were performed when mice were 8–12 weeks old. All behavioral tasks were performed during the light phase (11:00–17:00). Mice were transferred to the behavioral room 3 h before testing for acclimation. Propofol, etomidate, midazolam, dexmedetomidine, ropinirole, pramipexole, or bromocriptine was administered 10 min after fear memory retrieval, which consisted of a single 20 s Conditioned Stimulus (CS). For chemogenetic experiments, CNO was administered 10 min after CS, and propofol was injected 30 min after CNO administration.

Fear conditioning, retrieval, recall, extinction, renewal, and spontaneous recovery were conducted using Digbehav video‐recording software (Jiliang, Shanghai, China). Context A consisted of black‐walled chambers (17 cm × 17 cm × 25 cm) with stainless‐steel grid floors and was cleaned with 75% ethanol. Context B consisted of white‐walled chambers (17 cm × 17 cm × 25 cm) with white acrylic bottoms and was cleaned with 4% acetic acid. The experimental procedures were performed as previously described. Briefly, mice were handled and habituated to the experimental environment for 3 consecutive days and then allowed to acclimate to the conditioning chamber for 20 min. On day 1, mice underwent fear conditioning in Context A. Each mouse received five pure‐tone CS presentations (3500 Hz, 80 dB, 20 s), delivered at 120 s intervals. Each CS co‐terminated with a foot shock serving as an unconditioned stimulus (US; 0.5 mA, 2 s each). Mice were returned to their home cages 60 s after the final CS‐US pairing. On day 2, mice were placed in Context B and exposed to a single 20 s CS without foot shock to retrieve the established fear memory and initiate reconsolidation. On day 3, fear memory recall was assessed using four 20 s CS presentations delivered at 120 s intervals in Context B. Renewal and spontaneous recovery were assessed using the same CS presentation protocol as the recall test. Renewal was tested in Context A on day 4, whereas spontaneous recovery was assessed in Context B 28 days after renewal. For fear memory extinction, mice received 20 CS presentations (20 s each) in Context B without foot shock, with inter‐CS intervals of 30–120 s. During behavioral testing, the movement of the mice in context A and context B was recorded using a near‐infrared camera and analyzed by an experienced experimenter blinded to experimental group assignment. The time spent freezing during the tone was measured for each tone presentation, and the freezing duration in the 20 s period preceding the first cue was also calculated as the baseline freezing. Freezing was operationally defined as the complete absence of all somatic movement, with the exception of respiration.

For the Y maze test, mice were placed in one arm of the maze and allowed to explore all three arms freely for 5 min. Arm entries were recorded using an automated behavioral tracking system. Three consecutive choices of all three arms were regarded as a correct alternation. The percentage of spontaneous alternation was calculated as the number of alternations divided by the total number of arm entries minus 2, multiplied by 100. The apparatus was cleaned with 75% ethanol after each trial.

For the open field test (OFT), the open field chamber was made of transparent plastic (50 cm × 50 cm), and a 25 cm × 25 cm center square was auto‐defined by the behavioral software. Individual mice were placed in the center of the chamber, and their behavior was monitored for 5 min. Time spent in the center zone and total distance traveled were quantified as measures of anxiety‐like behavior and locomotor activity, respectively.

#### 5.2.6 Histology and c‐Fos Immunofluorescence

Mice were deeply anesthetized with tribromoethanol and transcardially perfused with saline followed by 4% paraformaldehyde (PFA). Brains were post‐fixed and cryoprotected in 30% sucrose prepared in 0.1 M phosphate‐buffered saline (PBS; pH 7.4) for 24–48 h. Coronal sections (40 µm) were prepared using a cryostat (Leica CM3050S). For general histological verification, sections were washed with 0.1 M PBS and counterstained with DAPI (4′,6‐diamidino‐2‐phenylindole) before mounting.

For c‐Fos immunostaining, mice underwent fear conditioning and fear memory retrieval and were perfused 90 min after treatment. Brain slices were blocked in PBS solution containing 10% bovine serum and 0.3% Triton X‐100, followed by incubation with a rabbit anti‐c‐Fos antibody (1:1000, Synaptic Systems #226008) for 48 h at 4°C. Then, brain slices were washed three times with 0.1 M PBS and incubated with a secondary antibody (donkey anti‐rabbit; Jackson ImmunoResearch, #711‐585‐152) for 12 h at room temperature. After washing, sections were mounted and counterstained with DAPI. c‐Fos‐positive cells were identified using a fluorescence‐intensity threshold set above background levels. Imaging parameters were kept constant across experimental groups, and automated cell counting was performed using QuPath 3.0. Fluorescence images were acquired using an Olympus VS120 microscope.

#### 5.2.7 Statistical Analyses

All statistical analyses were performed in GraphPad Prism 8.0 or MATLAB R2021a. Comparisons among multiple groups were performed using one‐way or two‐way ANOVA followed by Tukey's multiple‐comparisons test, as appropriate. Comparisons between two groups were performed using paired or unpaired Student's *t*‐tests, as appropriate. For datasets that did not meet parametric assumptions, the Kruskal–Wallis test followed by Dunn's multiple‐comparisons test was used. Differences were considered statistically significant at *p* < 0.05. Asterisks denote statistical significance ^*^ *p* < 0.05; ^**^ *p* < 0.01; ^***^ *p* < 0.001. Values are reported as mean ± SEM.

## Author Contributions

**Xiao‐Na Zhu**, **Ji Hu**, **Ti‐Fei Yuan**, and **Tao Xu** conceptualized the study and conducted the experimental design, investigation, and analyses. **Jie Li**, **Yi‐Ge Guo**, **Xing Xu, Han Li** and Gui‐Lin Meng performed the behavioral tasks and in vivo fiber photometry recording experiments. Xiao‐Na Zhu and Xing Xu performed the brain slice electrophysiology experiments. Yi‐Ge Guo performed the immunohistochemical experiments. Jie Li, **Gui‐Lin Meng**, and Xiao‐Na Zhu prepared the initial manuscript, which was further edited by Ji Hu, Ti‐Fei Yuan, Tao Xu, and Xiao‐Na Zhu. Jie Li, **Xin‐Yuan Chen**, Yi‐Ge Guo, Xing Xu, Gui‐Lin Meng, **Liu‐Wei Chu, and Xiao ‐ Yun Guo** conducted data presentation and visualization. Xiao‐Na Zhu, Ji Hu, Ti‐Fei Yuan, and Tao Xu were responsible for project administration. Ji Hu, Ti‐Fei Yuan, and Xiao‐Na Zhu supervised the research. Xiao ‐ Yun Guo, Jie Li, Xing Xu, and Xiao‐Na Zhu acquired funding.

## Conflicts of Interest

The authors declare no conflicts of interest.

## Supporting information




**Supporting File**: advs78527‐sup‐0001‐SuppMat.pdf.

## Data Availability

The data that support the findings of this study are available on request from the corresponding author. The data are not publicly available due to privacy or ethical restrictions.
