# Supplementary material for: Propofol Modulates Fear Memory Reconsolidation by Inhibiting D2 Receptor‐Expressing Neurons in the Medial Prefrontal Cortex
Source: Adv Sci (Weinh). 2026 Oct 28:e78527. Online ahead of print. doi: 10.1002/advs.78527 (PMC13717115; doi:10.1002/advs.78527)
Supplement: Supplementary file 1 [file ADVS-9999-e78527-s001.pdf]

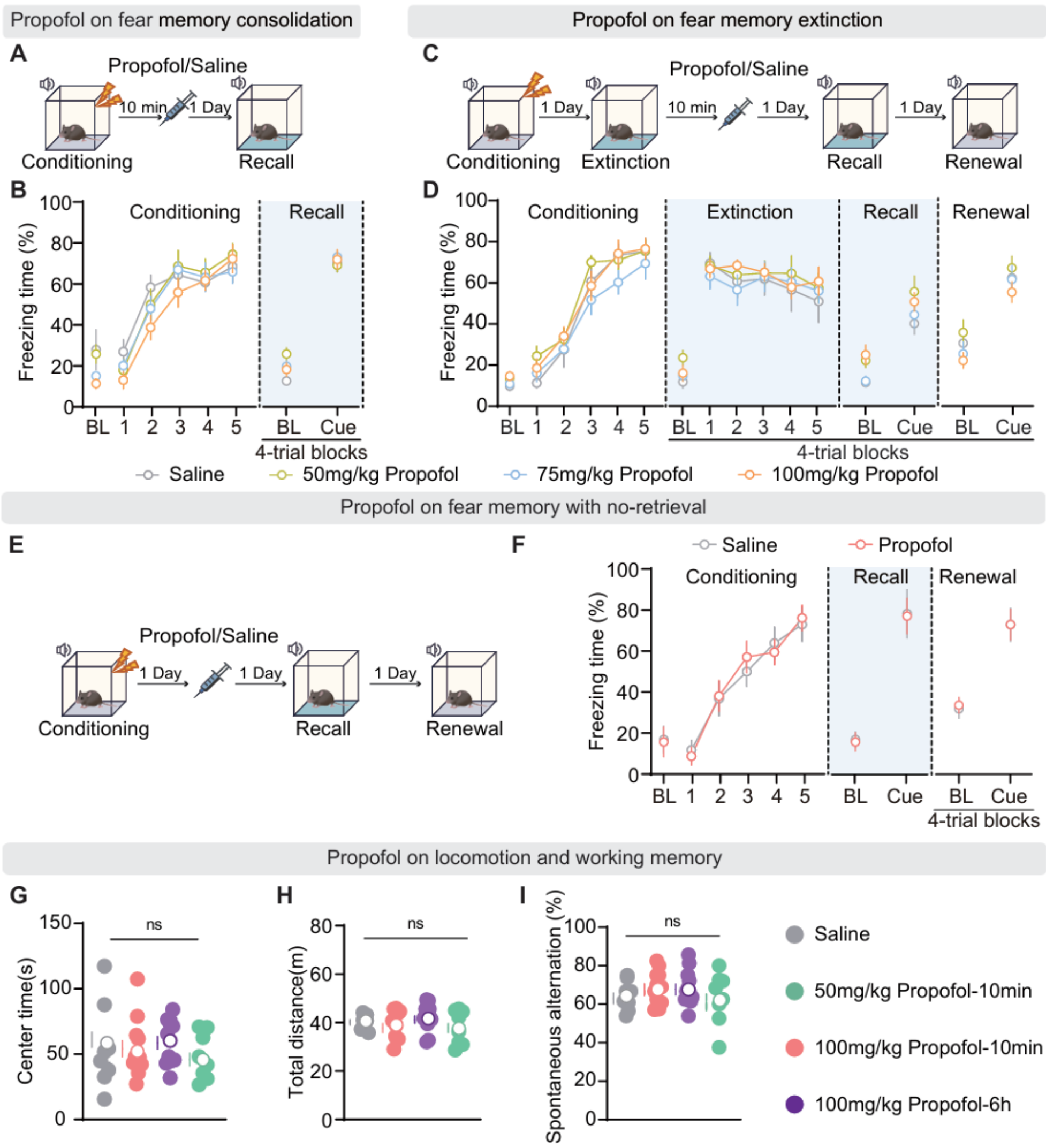

Figure S1. The effects of propofol on fear memory consolidation, extinction, locomotion, and working memory, Related to Figure 1

(A) Schematic diagram of the behavioral experimental protocol.

(B) Freezing responses to the context alone (baseline) or to the CS in saline or propofol treated mice during fear conditioning and recall. Statistics were as follows: one-way ANOVA followed by Tukey's test; condition, p =0.5314; recall test, p = 0.7365 (n = 8 mice per group).

(C) Schematic diagram of the behavioral experimental protocol.

(D) Freezing responses to the context alone (baseline) or to the CS in saline or propofol treated mice during fear conditioning, extinction, recall, and renewal. Statistics were as follows: one-way ANOVA followed by Tukey's test; recall test, p = 0.8124; renewal test, p =0.6534 (n = 8 mice per group).

(E) Schematic diagram of the behavioral experimental protocol without fear memory retrieval before propofol or saline administration.

(F) Freezing responses to the context alone (baseline) or to the CS in saline or propofol treated mice in the absence of prior memory retrieval, during fear conditioning,

recall, and renewal. Statistics were as follows: unpaired Student's t-test; recall test, $p = 0.6936$; renewal test, $p = 0.8526$ ($n = 8$ mice per group).

(G) Time spent in the center zone during the open-field test in saline and propofol treated mice ($n = 8$-12 mice per group).

(H) Total distance traveled during the open-field test in saline and propofol treated mice ($n = 8$-12 mice per group).

(I) Spontaneous alternation percentage in the Y-maze test in saline and propofol-treated mice ($n = 8$-12 mice per group).

Statistical significance was determined by unpaired Student's t-test or one-way ANOVA followed by Tukey's multiple-comparisons test, as appropriate. Data are presented as the mean ± SEM.

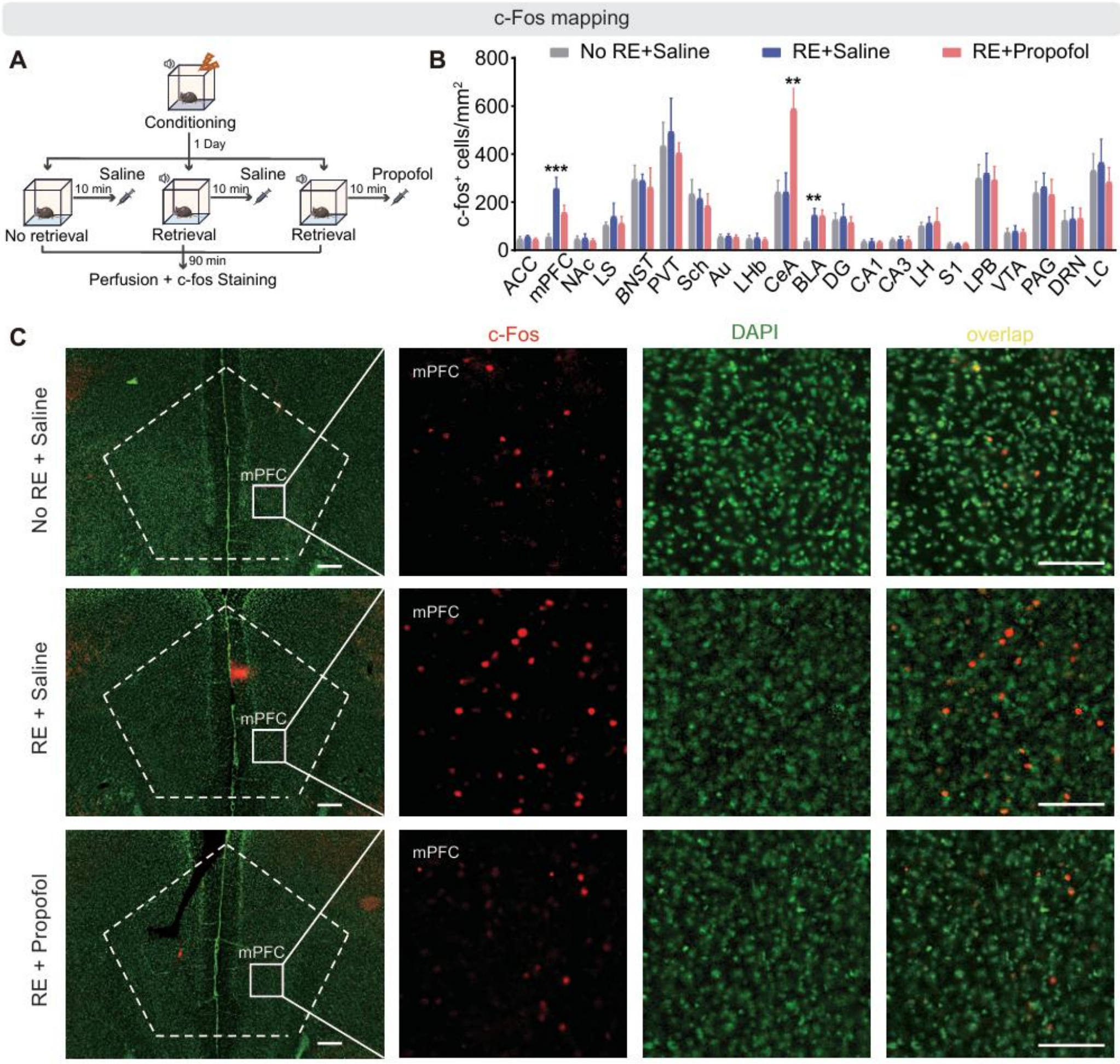

c-Fos mapping
A
Conditioning
1 Day
Saline
10 min
Saline
10 min
Propofol
10 min
No retrieval
Retrieval
Retrieval
90 min
Perfusion + c-fos Staining
B
No RE+Saline
RE+Saline
RE+Propofol
c-fos+ cells/mm2
800
600
400
200
0
***
**
**
ACC
mPFC
NAc
LS
BNST
PVT
Sch
Au
LHb
CeA
BLA
DG
CA1
CA3
LH
S1
LPB
VTA
PAG
DRN
LC
C
c-Fos
DAPI
overlap
No RE + Saline
RE + Saline
RE + Propofol
mPFC

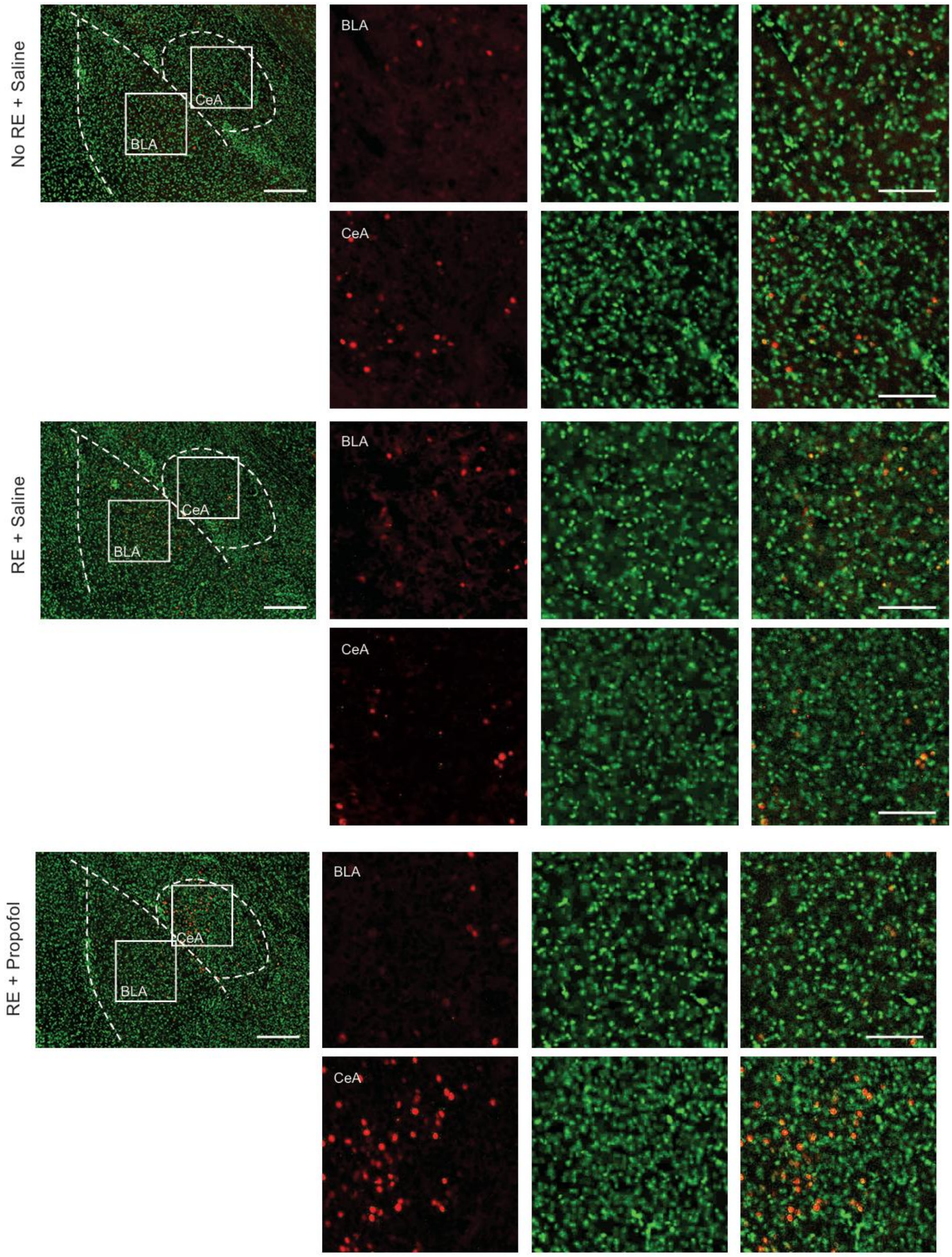

Figure S2. Propofol decreases reconsolidation-associated c-Fos activation in the mPFC, Related to Figure 2

(A) Schematic diagram of the c-Fos mapping protocol.

(B) Quantification of c-Fos-positive cell density across the indicated brain regions in no-retrieval + saline, retrieval + saline, and retrieval + propofol groups (n = 4-6 mice per group).

(C) Representative images of c-Fos (red) and DAPI (green) immunofluorescence in the mPFC, BLA, and CeA from no-retrieval + saline, retrieval + saline, and retrieval + propofol groups. Merged images are shown in the right column. Overview (left), scale bar, 250 μm; magnified view (right), scale bar, 100 μm

Statistical significance was determined by two-way ANOVA followed by Tukey's multiple-comparisons test. Data are presented as the mean ± SEM. $^{**}p < 0.01$; $^{***}p < 0.001$.

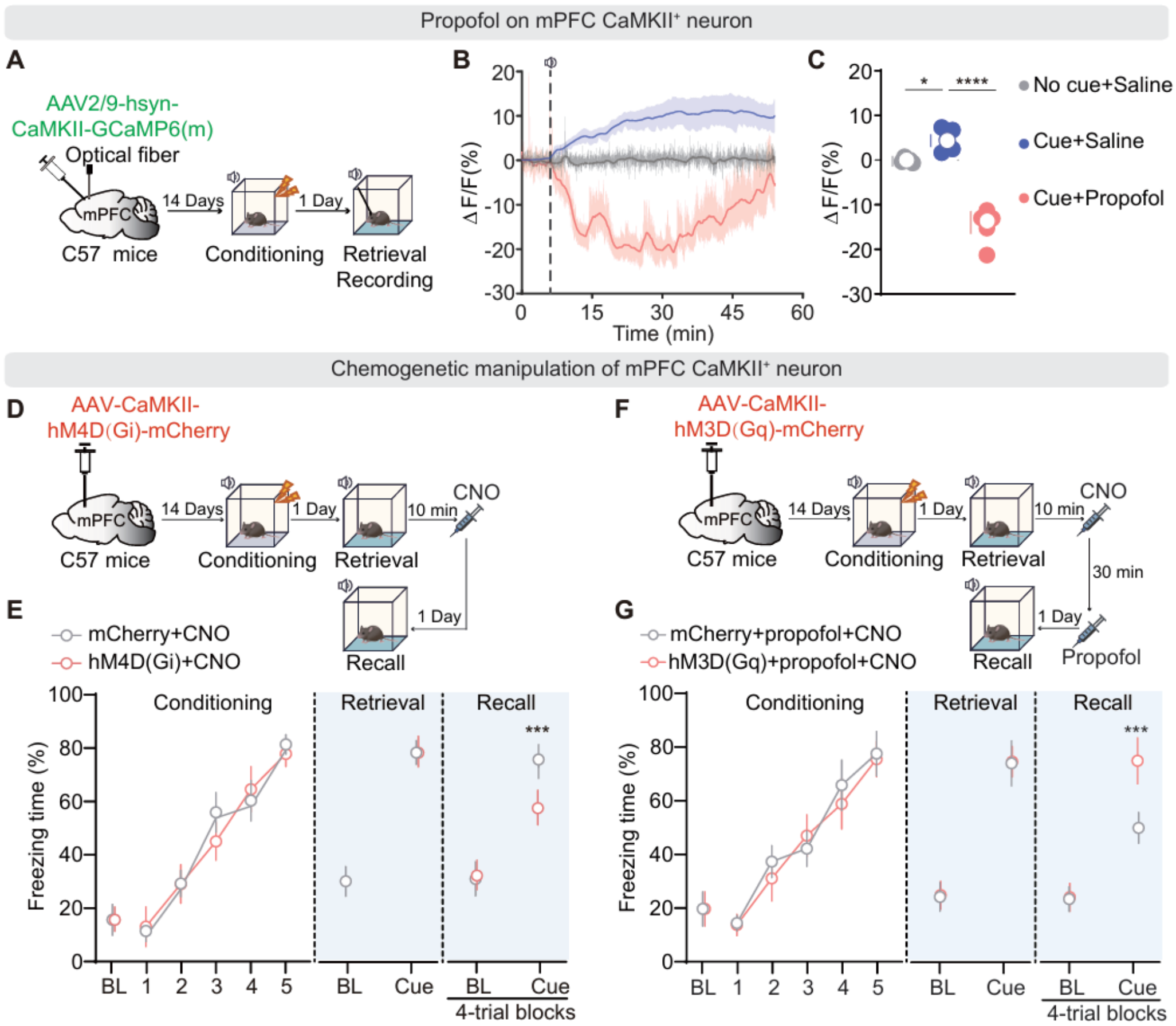

Figure S3. Propofol inhibits mPFC CaMKII$^+$ neurons to modulate fear memory reconsolidation, Related to Figure 2

(A) Schematic of fiber implantation over the mPFC following expression of GCaMP6m in CaMKII$^+$ neurons of C57BL/6J mice.

(B) GCaMP6m signals from mPFC CaMKII$^+$ neurons aligned to the onset of CS presentation in the no CS + saline, CS + saline, and CS + propofol (100mg/kg) groups.

(C) Quantification of the changes in GCaMP6m signals from mPFC CaMKII$^+$ neurons in C57BL/6J mice following no CS+saline, CS+saline, and CS+propofol (100mg/kg) (n = 5 mice per group).

(D) Schematic diagram of chemogenetic inhibition of mPFC CaMKII$^+$ neurons and the behavioral experimental protocol.

(E) Freezing responses to the context alone (baseline) or to the CS in mice expressing mCherry or hM4D(Gi) in mPFC CaMKII$^+$ neurons. Statistics were as follows: unpaired Student's t-test; retrieval test, $p = 0.8421$; recall test, $p = 0.0005$ (n = 8 mice per group).

(F) Schematic diagram of chemogenetic activation of mPFC CaMKII$^+$ neurons during propofol treatment and the behavioral experimental protocol.

(G) Freezing responses to the context alone (baseline) or to the CS in propofol-treated mice expressing mCherry or hM3D(Gq) in mPFC CaMKII$^+$ neurons. Statistics are

as follows: unpaired Student's t-test; retrieval test, $p = 0.7659$; recall test, $p = 0.0007$ ($n = 8$ mice per group).

Statistical significance was determined by one-way ANOVA followed by Tukey's multiple-comparisons test or unpaired Student's t-test, as appropriate. Data are presented as the mean ± SEM. *$p < 0.05$; ***$p < 0.001$; ****$p < 0.0001$

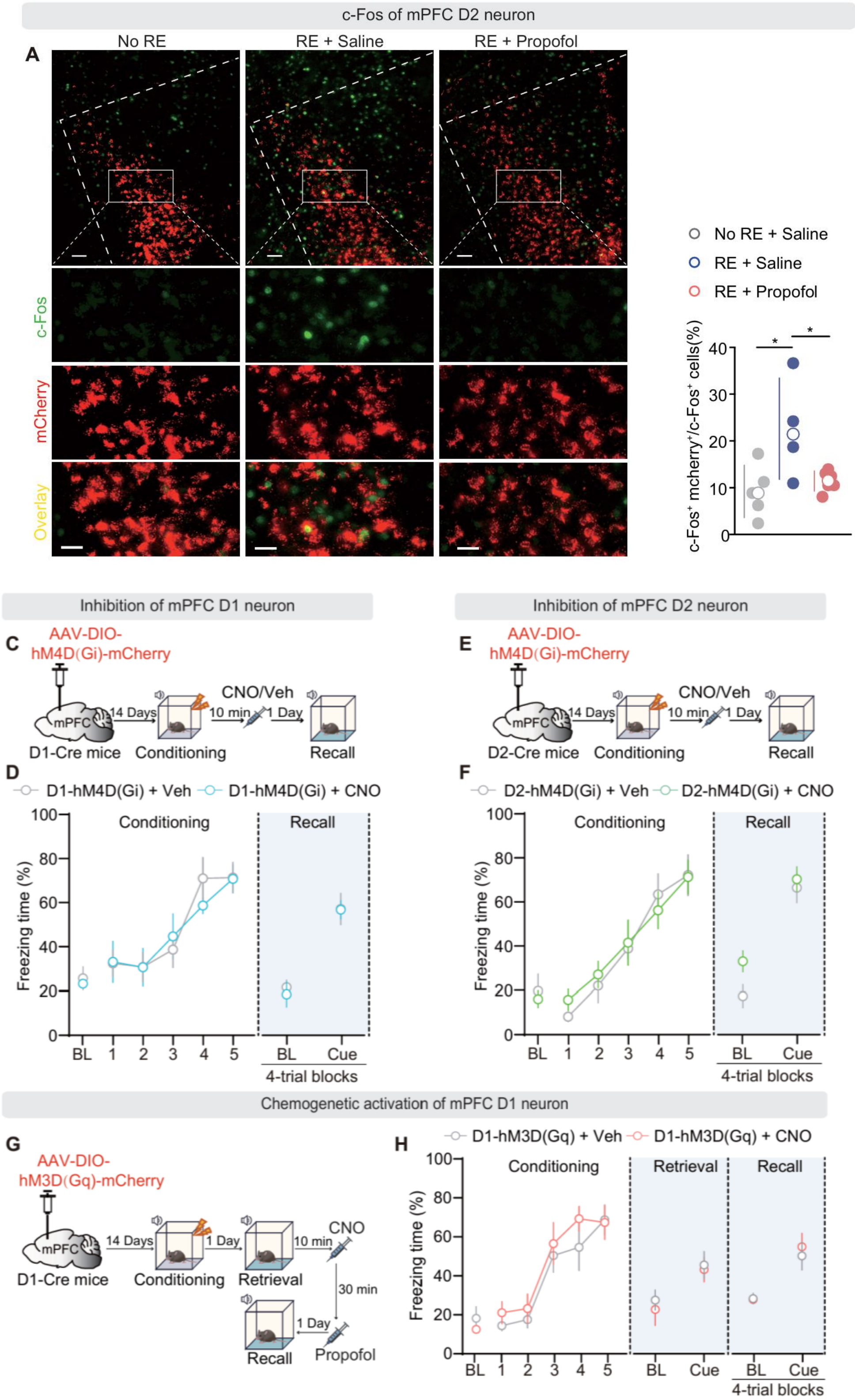

Figure S4. Role of mPFC D1 and D2 neurons in fear memory reconsolidation, Related to Figure 2

(A) Representative images of c-Fos (green) immunofluorescence in mPFC D2^{+}

neurons from control, fear memory retrevial, and propofol-treated mice. Scale bar, 50 μm (top) and 25 μm (bottom).

(B) Quantification of the proportion of c-Fos^+/$mCherry^+$ double-positive cells among total c-Fos^+ cells in the mPFC of control, fear memory retrieval, and propofol-treated mice (n = 4-6 mice per group).

(C) Schematic diagram of the behavioral experimental protocol without memory retrieval.

(D) Freezing responses to the context only (baseline) or to the CS in vehicle- and CNO-treated mice expressing hM4D(Gi) in mPFC D1 neurons, tested in the absence of prior memory retrieval. Statistics are as follows: unpaired Student's t-test; recall test, $p = 0.9348$ (n = 8 mice per group).

(E) Schematic diagram of behavioral experimental protocol without memory retrieval.

(F) Freezing responses to the context alone (baseline) or to the CS in vehicle- and CNO-treated mice expressing hM4D(Gi) in mPFC D2 neurons, tested in the absence of prior memory retrieval. Statistics were as follows: unpaired Student's t-test; recall test, $p = 0.6751$ (n = 8 mice per group).

(G) Schematic diagram of the behavioral experimental protocol.

(H) Freezing responses to the context alone (baseline) or to the CS in vehicle and CNO treated mice expressing hM3D(Gq) in mPFC D1 neurons during propofol treatment. Statistics were as follows: unpaired Student's t-test; retrieval test, $p =0.7634$; recall test, $p =0.8372$ (n = 8 mice per group).

Statistical significance was determined by one-way ANOVA followed by Tukey's multiple-comparisons test or unpaired Student' s t-test. Data are presented as the mean ± SEM. $*p < 0.05$.

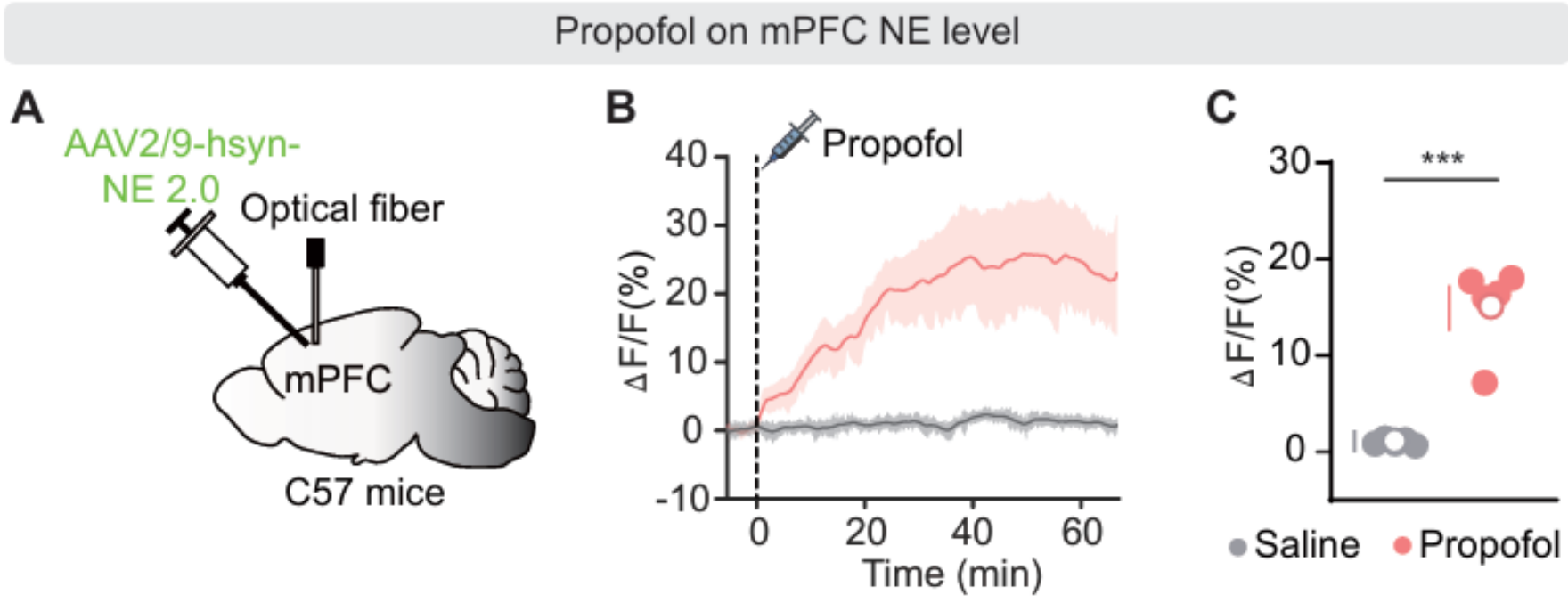

Figure S5. Effect of propofol on mPFC NE levels, Related to Figure 3

(A) Schematic of fiber implantation over the mPFC following expression of the NE2.0 sensor in C57BL/6J mice.

(B) NE sensor signals from the mPFC aligned to the time of saline or propofol administration (100 mg/kg).

(C) Quantification of changes in NE sensor signals after administration of saline or propofol (100mg/kg) in the mPFC of C57BL/6J mice (n = 5 mice per group).

Statistical significance was determined by unpaired Student' s t-test. Data are presented as the mean ± SEM. ***$p < 0.001$.

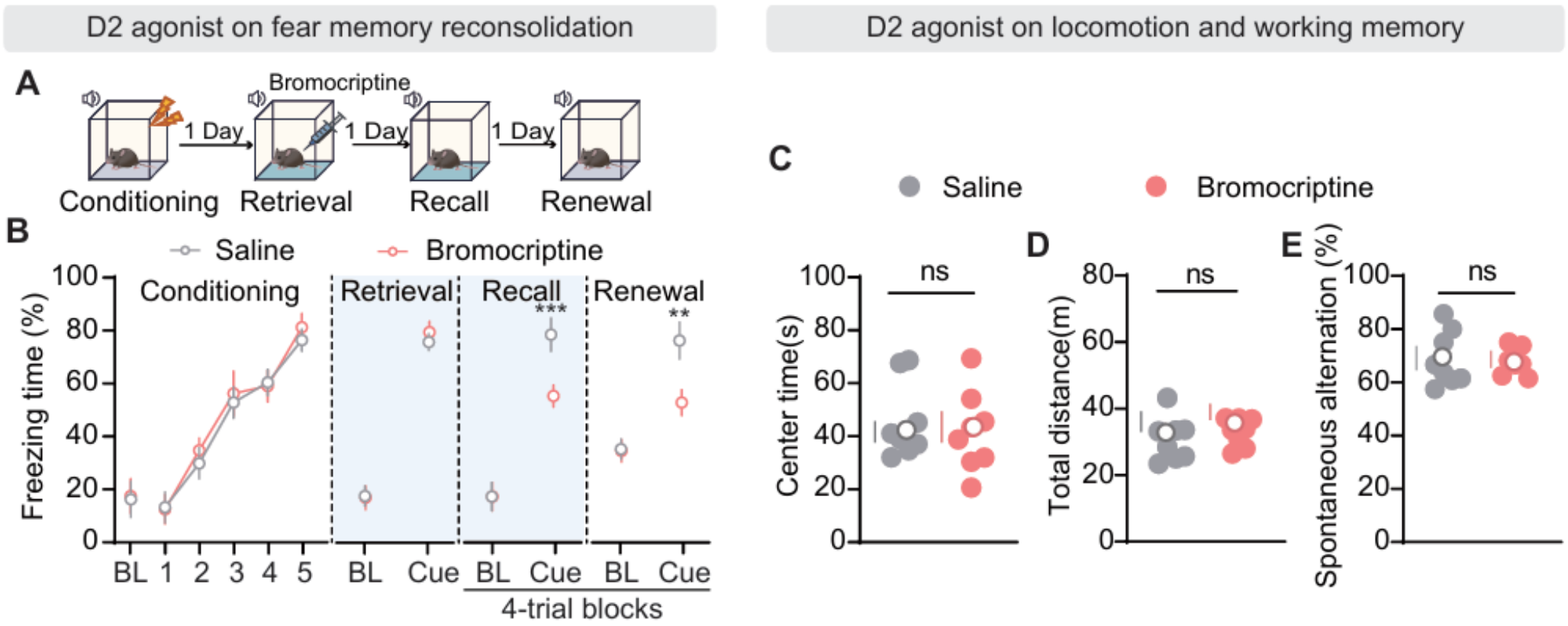

Figure S6. Effects of bromocriptine on fear memory, locomotor activity, and working memory, Related to Figure 3

(A) Schematic diagram of the behavioral experimental protocol.

(B) Freezing responses to the context alone (baseline) or to the CS in saline and bromocriptine-treated mice. Statistics were as follows: unpaired Student's t-test; retrieval test, $p = 0.4724$; recall test, $p = 0.0003$; renewal test, $p = 0.0024$ (n = 8-12 mice per group).

(C) Time spent in the center zone during the open-field test in saline and bromocriptine treated mice (n = 8-12 mice per group).

(D) Total distance traveled during the open-field test in saline and bromocriptine treated mice (n = 8-12 mice per group).

(E) Spontaneous alternation percentage in the Y-maze test in saline and bromocriptine-treated mice (n = 8-12 mice per group).

Statistical significance was determined by unpaired Student's t-test. Data are presented as the mean ± SEM. $^{**}p < 0.01$. $^{***}p < 0.001$.

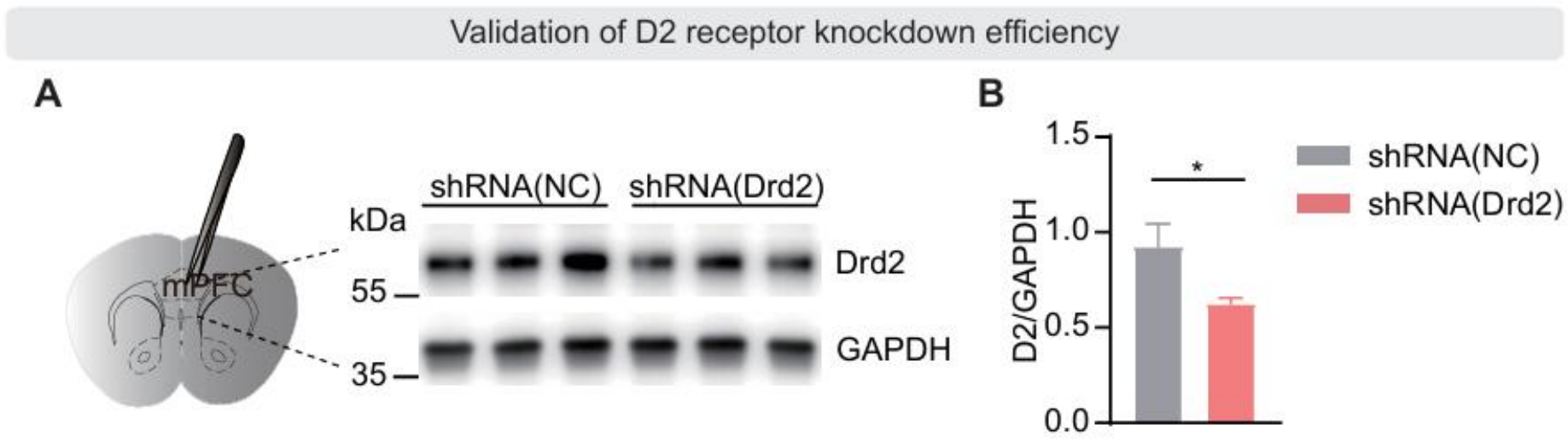

Figure S7. Validation of shRNA-mediated D2 receptor knockdown in the mPFC, Related to Figure 4

(A) Representative western blot showing shRNA-mediated knockdown of D2 receptor expression in the mPFC.

(B) Quantification of D2 receptor protein levels normalized to GAPDH in the mPFC of control shRNA- and Drd2-targeting shRNA-treated mice.

Statistical significance was determined using an unpaired Student’s t-test. Data are presented as the mean ± SEM. *$p < 0.05$ (n=3 mice per group).

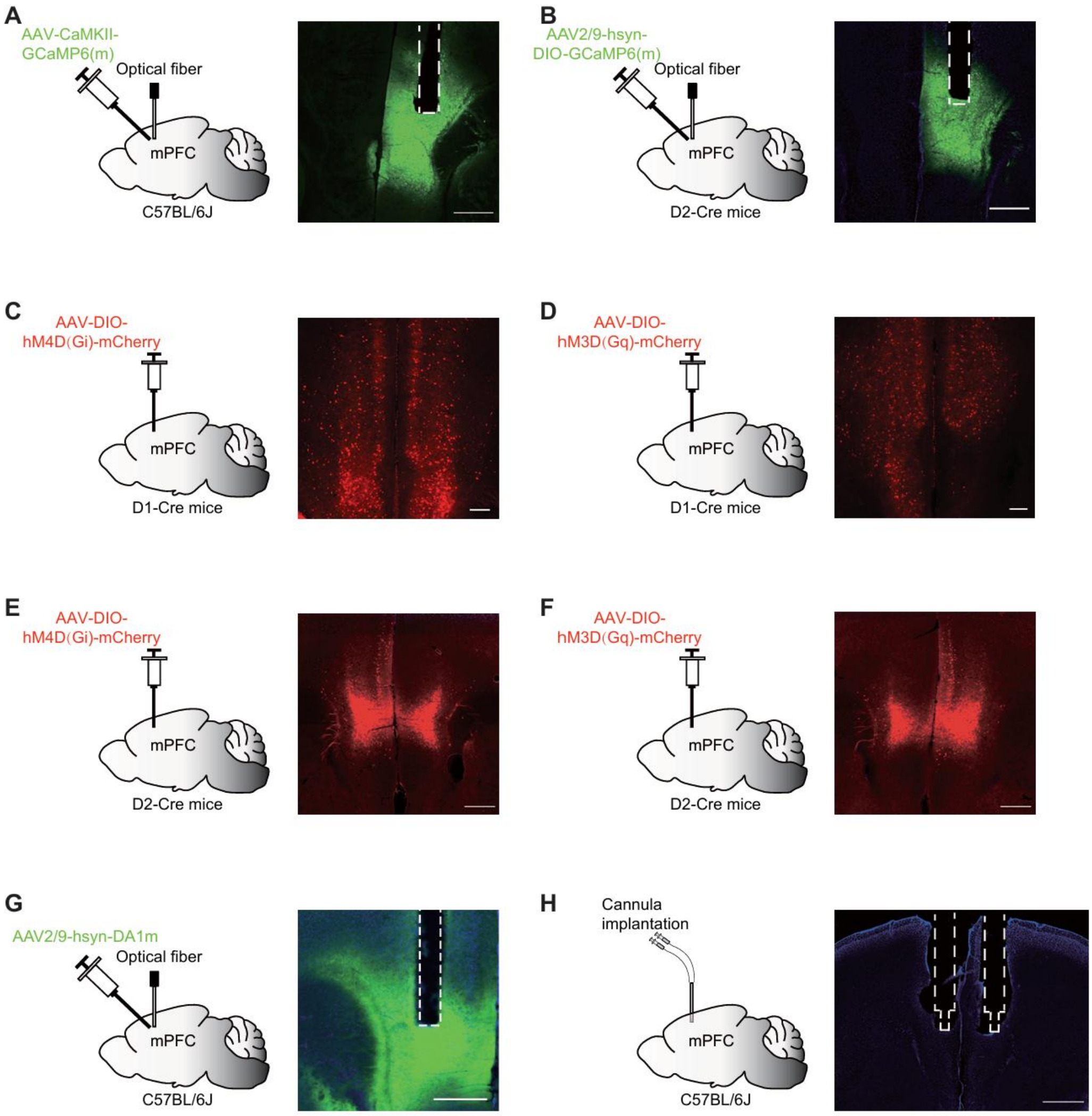

Figure S8. Histological verification of viral expression and the locations of optical fibers and cannulae, Related to Figures 2-3

(A) Histological verification of AAV2/9-hSyn-CaMKII-GCaMP6m expression and optical fiber placement in the mPFC of C57BL/6J mice. Scale bar, 400 μm.

(B) Histological verification of AAV2/9-hsyn-DIO-GCaMP6m expression and optical fiber placement in the mPFC of D2-Cre mice. Scale bar, 400 μm.

(C) Histological verification of AAV-DIO-hM4D(Gi)-mCherry expression in the mPFC of D1-Cre mice. Scale bar, 200 μm.

(D) Histological verification of AAV-DIO-hM3D(Gq)-mCherry expression in mPFC of D1-Cre mice. Scale bar, 200 μm.

(E) Histological verification of AAV-DIO-hM4D(Gi)-mCherry expression in mPFC of D2-Cre mice. Scale bar, 400 μm.

(F) Histological verification of AAV-DIO-hM3D(Gq)-mCherry expression in mPFC of D2-Cre mice. Scale bar, 400 μm.

(G) Histological verification of AAV-hSyn-DA1m-WPRE-PA expression and optical fiber placement in the mPFC of C57BL/6J mice. Scale bar, 400 μm.

(H) Histological verification of the cannula placement in the mPFC of C57BL/6J mice. Scale bar, 1000 μm.
